# Conservation of the genome-wide recombination rate in white-footed mice

**DOI:** 10.1038/s41437-019-0252-9

**Published:** 2019-07-31

**Authors:** April L. Peterson, Nathan D. Miller, Bret A. Payseur

**Affiliations:** 1 Laboratory of Genetics, University of Wisconsin–Madison, Madison, WI 53706 USA; 2 Department of Botany, University of Wisconsin–Madison, Madison, WI 53706 USA

**Keywords:** Evolutionary biology, Eukaryote

## Abstract

Despite being linked to the fundamental processes of chromosome segregation and offspring diversification, meiotic recombination rates vary within and between species. Recent years have seen progress in quantifying recombination rate evolution across multiple temporal and genomic scales. Nevertheless, the level of variation in recombination rate within wild populations—a key determinant of evolution in this trait—remains poorly documented on the genomic scale. To address this notable gap, we used immunofluorescent cytology to quantify genome-wide recombination rates in males from a wild population of the white-footed mouse, *Peromyscus leucopus*. For comparison, we measured recombination rates in a second population of male *P. leucopus* raised in the laboratory and in male deer mice from the subspecies *Peromyscus maniculatus bairdii*. Although we found differences between individuals in the genome-wide recombination rate, levels of variation were low—within populations, between populations, and between species. Quantification of synaptonemal complex length and crossover positions along chromosome 1 using a novel automated approach also revealed conservation in broad-scale crossover patterning, including strong crossover interference. We propose stabilizing selection targeting recombination or correlated processes as the explanation for these patterns.

## Introduction

Meiotic recombination is one of the main sources of genetic variation. Recombination can facilitate or hinder adaptation by disrupting deleterious or beneficial combinations of alleles at different loci and by changing additive genetic variance (Hill and Robertson 1966; Barton 1995; Charlesworth and Barton 1996; Stapley et al. 2017). In virtually all species that reproduce through sex, recombination is critical to the proper segregation of homologous chromosomes during meiosis (Hassold and Hunt 2001). Despite the evolutionary and genetic significance of crossing over, the rate at which it occurs varies between and within closely related species (Smukowski and Noor 2011; Dapper and Payseur 2017; Ritz et al. 2017; Stapley et al. 2017).

Natural variation in recombination rate among individuals within populations is the substrate for the evolution of this fundamental genomic parameter. Linkage maps have uncovered inter-individual variation in the total number of crossovers across the genome (the genome-wide recombination rate)—as well as genetic variants associated with these differences—within populations of humans (Kong et al. 2004, 2008; Halldorsson et al. 2019), domesticated cattle (Sandor et al. 2012; Ma et al. 2015), and domesticated sheep (Davenport et al. 2018). Despite this progress, the extent of variation in genome-wide recombination rate within wild populations remains poorly understood (but see Gion et al. 2016; Johnston et al. 2016).

The genome-wide recombination rate is a promising focus for understanding natural variation in crossing over. The total number of crossovers is intrinsically connected to meiotic constraints, including lower and upper thresholds that reduce fertility and offspring viability when exceeded (Hassold and Hunt, 2001; Ritz et al. 2017). When the number of crossovers is very low, the incidence of aneuploidy can increase substantially (Hassold and Hunt 2001), thereby reducing individual and population fitness. The minimum threshold appears to range between one crossover per chromosome and one crossover per chromosome arm, at least in mammals (Dumont 2017). Although the fitness effects of excessive recombination remain poorly defined, the existence of an upper bound seems likely. Too many crossovers could elevate the frequency of aneuploidy (Koehler et al. 1996), though artificial increases in recombination rate did not increase rates of non-disjunction in *Arabidopsis thaliana* (Serra et al. 2018; Fernandes et al. 2018). Another potential contributor to an upper ceiling on crossover number is DNA damage. Each crossover originates as a double-strand break (DSB), but meiotic cells typically generate many more DSBs than crossovers (Anderson and Stack 2005; Chelysheva et al. 2007; Giraut et al. 2011). There is evidence from humans and yeast that a large number of DSBs are repaired as non-crossovers through trans-lesion synthesis by DNA polymerase θ, a process that is more mutagenic than ordinary DNA synthesis (Hogg et al. 2011; Arbel-Eden et al. 2013). Together, lower and upper bounds are expected to impose stabilizing selection on the genome-wide recombination rate in natural populations.

Another advantage of focusing on the genome-wide recombination rate is that it can be estimated in single meiotic cells, enabling improved characterization of the process of crossing over at the level of individuals. The genome-wide recombination rate has been quantified in a broad range of species by counting chiasmata from metaphase spreads (e.g. Burt et al. 1991). This approach has been largely replaced with immunocytology using the mismatch repair protein, MLH1, as a marker for mature crossovers (Anderson et al. 1999).

Since it does not require crosses or identification of relatives (in contrast to linkage maps), the strategy of counting MLH1 foci is well-suited for quantifying genome-wide recombination rate in wild individuals. Although it cannot detect a minority of non-interfering (type II) crossovers formed through an alternative pathway (~10% in mammals; Holloway et al. 2008), the MLH1 approach has substantially expanded the number of species with recombination rate estimates (especially in mammals and birds (Stapley et al. 2017) and several plant species (Lhuissier et al. 2007; Phillips et al. 2015)). Notably, this powerful method has seldom been used to profile genome-wide recombination rate in more than a few individuals from a species (but see Ruiz-Herrera et al. 2017), again leaving the level of natural variation in this fundamental meiotic trait poorly documented.

The MLH1 approach depends on visualizing the synaptonemal complex (SC) to identify crossovers and estimate cellular timing within prophase. During meiosis, homologous chromosomes are arranged into structured arrays of tethered loops and joined by the SC when they undergo synapsis (Moses 1968). When the physical amount of DNA (in base pairs) is relatively constant, SC length and chromatin loop size are interdependent variables (Kleckner et al. 2003; Novak et al. 2008). This aspect of chromosome organization in meiosis has also been proposed to modulate DSB formation and crossover number (Lynn et al. 2002; Kleckner et al. 2003; Ruiz-Herrera et al. 2017). Therefore, measuring inter-individual variation in SC length and MLH1 foci position along pachytene bivalents provides useful information about a potential determinant of recombination rate and reveals sub-chromosomal locations of crossovers.

The main aim of this manuscript is to characterize variation in recombination rate among individuals from a natural population. We used immunocytology to quantify variation in genome-wide recombination rate among wild males of the white-footed mouse, *Peromyscus leucopus*. *Peromyscus* is one of the most speciose and abundant groups of mammals in North America, making it an excellent model for understanding the evolution of a wide variety of phenotypes (Bedford and Hoekstra 2015). To provide context for the patterns of variation we document in the wild, we measured genome-wide recombination rates from multiple *P. leucopus* and *P. maniculatus bairdii* males raised in a common laboratory environment. We found that the genome-wide recombination rate is highly constrained, both within and between species. The heterogeneous structure of the *P. leucopus* karyotype enabled us to isolate and characterize SC length and crossover patterning along a single bivalent (chromosome 1) using a novel, automated image analysis tool. We found more variation in SC length in wild mice than in laboratory mice, whereas broad-scale crossover patterning was similar. Our results provide a rare portrait of natural variation in the genome-wide recombination rate.

## Materials and methods

### Mice

To measure recombination rate variation within a wild population, we caught 23 *Peromyscus leucopus* from nine trapping sites in Madison, Wisconsin, between August 2017 and July 2018. To minimize effects of population structure, we selected trapping sites within an approximately one mile radius centered on the University of Wisconsin-Madison campus. Sites included agricultural buildings, public outdoor spaces, and residences. At each site, 12 Sherman traps baited with peanut butter and oats were left overnight. Traps were checked the next morning. Mice were euthanized by cervical dislocation and sexed by inspection of genital morphology. To confirm species identity, we sequenced two stretches of mitochondrial D-Loop, from 486 to 921 bp and from 5347 to 15,799 bp in the reference sequence (using primers DL-PeroF with DL-PeroR and L15926 with H16340, respectively, from Fiset et al. 2015). We PCR-amplified these loci using the conditions and primers from Fiset et al. (2015). PCR products were sequenced using Sanger chemistry on an ABI 3730xl DNA Analyzer at the University of Wisconsin-Madison Biotechnology Sequencing Facility. Raw sequence files were viewed in FinchTV (v1.4.0), and then aligned in MEGA (v10.0.4) using CLUSTALW with default settings. Sequences were BLASTed on NCBI. The best hits for all sequences from the 23 mice were *Peromyscus leucopus*. All subsequent work on this sample focused on a total of nine males from six trapping sites, for which we could examine recombination in meiotic cells (Supplementary Table 1).

To provide context and comparisons for recombination rate variation in wild *P. leucopus*, we surveyed two additional groups of mice. Ten male *P. leucopus* and three male *P. m. bairdii* were obtained from the Peromyscus Stock Center (PSC; University of South Carolina). The *P. leucopus* (“LL”) were from an outbred colony established in 1982 by mating 38 mice caught near Linville, North Carolina, USA (https://www.pgsc.cas.sc.edu/peromyscus-leucopus-white-footed-mouse-ll-stock). *The P. m. bairdii* (“BW”) were from an outbred colony established in 1946 by mating 40 mice caught near Ann Arbor, Michigan (https://www.pgsc.cas.sc.edu/peromycus-maniculatus-deer-mouse-bw-stock). The 13 mice we surveyed were raised in a common laboratory environment at the PSC with a similar age range (Supplemental Table 2), suggesting that differences in recombination rate among them are genetic. Mice were euthanized by CO_2_ asphyxiation upon arrival at the University of Wisconsin -Madison. All work followed protocols approved by the Institutional Animal Care and Use Committee at the University of Wisconsin-Madison.

### Tissue collection and immunohistochemistry

Spermatocyte spreads were prepared following Peters et al. (1997) with minor adjustments. The tunica albuginea was removed and whole testis was incubated in 3 ml of hypotonic solution for 45 min. The incubated testis was transferred to 40 μl of 100 mM sucrose on a microscope slide and torn with fine forceps. Approximately 15 μl of cell slurry added to 80 μl of a 2% PFA solution was spread onto a glass slide and dried overnight in a humid chamber. Immunohistochemistry followed Anderson et al. (1999) and Koehler et al. (2002). Antibody work and slide blocking were conducted in 1× antibody dilution buffer (ADB), normal donkey serum (Jackson ImmnuoResearch, West Grove PA, USA), 1× phosphate-buffered saline (PBS), and bovine serum albumin (Sigma-Aldrich, St. Louis, MO, USA), Triton X-100 (Sigma-Aldrich, St. Louis, MO, USA). Each slide was blocked for 30 min in ADB before 60 μl of a primary antibody mix containing rabbit anti-MLH1 polyclonal antibody to MLH1 (Calbiochem, San Diego, CA, USA; diluted 1:50), anti-goat polyclonal antibody to human SYCP3 (R&D Systems, Minneapolis, MN, USA; diluted 1:50), and anti-human polyclonal antibody to CREST (Antibodies Inc., Davis, CA, USA; diluted 1:200) in ADB was incubated for 48 h at 37°. Slides were washed twice in 50 ml ADB between primary and secondary antibody incubations. Slides were incubated overnight at 37° in Alexa Fluor 488 donkey anti-rabbit IgG (Invitrgoen, Carlsbad, CA, USA; diluted to 1:100) and Coumarin AMCA donkey anti-human IgG (Jackson ImmunoResearch, West Grove PA, USA; diluted to 1:200). Alexa Fluor 568 donkey anti-goat (Invitrogen, Carlsbad, CA, USA; diluted 1:100) was incubated at 1:100 for 2 h at 37°. Slides were washed in 1× PBS, dried, and fixed with Prolong Gold Antifade (Invitrogen, Carlsbad, CA, USA) for at least 24 h. Mice with at least 10 cells with good staining were included in our analysis. Due to variable quality of spermatocyte spreads four mice were used for quantification of either MLH1 counts or chromosome 1 SC traits instead of both (Supplemental Tables 1 and 2).

### Imaging, recombination rate, and synaptonemal complex length

Spermatocytes were imaged using a Zeiss Axioplan 2 microscope with AxioLab camera and Axio Vision software (Zeiss, Cambridge, UK). Preprocessing, including cropping, noise reduction, and histogram adjustments, was performed using Photoshop (v13.0). Only cells with a full karyotype (23 autosomes, 1XY), intact bivalents, and clear, distinct MLH1 foci were included for quantification. Generally, foci size and intensity were consistent across bivalents within the same nucleus. However, closer examination of chromosome 1 bivalents revealed smaller foci which could be distinguished from the background foci by higher intensity and placement centered within the width of the SC. Image file names were anonymized before manual scoring. We recorded the numbers of MLH1 foci, bivalents with 0 MLH1 foci, and bivalents with signs of asynapsis. We also recorded a quality score (ranging from 1 to 5, with 1 representing high quality), whether or not the X and Y were paired, and whether or not a MLH1 focus was present in the pseudo-autosomal region on the X and Y as quality control measures.

To automate the quantitative measurement of single bivalents, we developed the DNACrossover method within the Phytomorph ToolKit on CyVerse. The Phytomorph toolkit is a collection of 20 high through-put image-analysis tools developed for plant phenotyping (reviewed in Spalding and Miller, 2013). To our knowledge, DNACrossover is the only algorithm which can isolate and measure single bivalents from images of pachytene stage meiocytes. Previous studies using single bivalent measures have been performed by manually tracing over image files using software such as Fiji/ImageJ, Micromeasure, or Photoshop. Those who wish to use DNACrossover can create a CyVerse account (https://de.cyverse.org/de/) and contact the authors who will provide access to the tool.

**Fig. 1:**
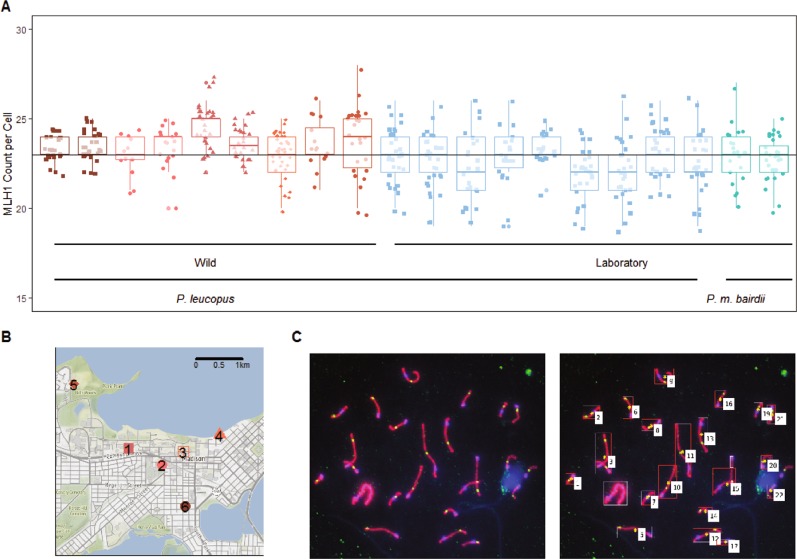
**a** Distributions of MLH1 focus counts with a small amount of noise added so points do not overlap. Each point represents a single spermatocyte; each column represents one mouse. Boxplots overlaid to illustrate mouse means. Red = wild *P. leucopus* from Madison, Wisconsin; light blue = *P. leucopus* from the Peromyscus Stock Center; green = *P. m. bairdii* from the Peromyscus Stock Center. Horizontal line (at 23) denotes the haploid number of autosomes per cell. **b** Map of trapping sites in Madison, Wisconsin. Colors match those in **a**. **c** Representative pachytene spermatocyte from a *P. leucopus* and whole-cell output file from automated method. Cells are stained for SYCP3, which forms part of the lateral element of the synaptonemal complex (red). Sites of recombination within the synaptonemal complex are marked by MLH1 (green), and the centromere is marked by CREST (blue). Algorithm-identified chromosomes are marked by a red box and index number

**Fig. 2:**
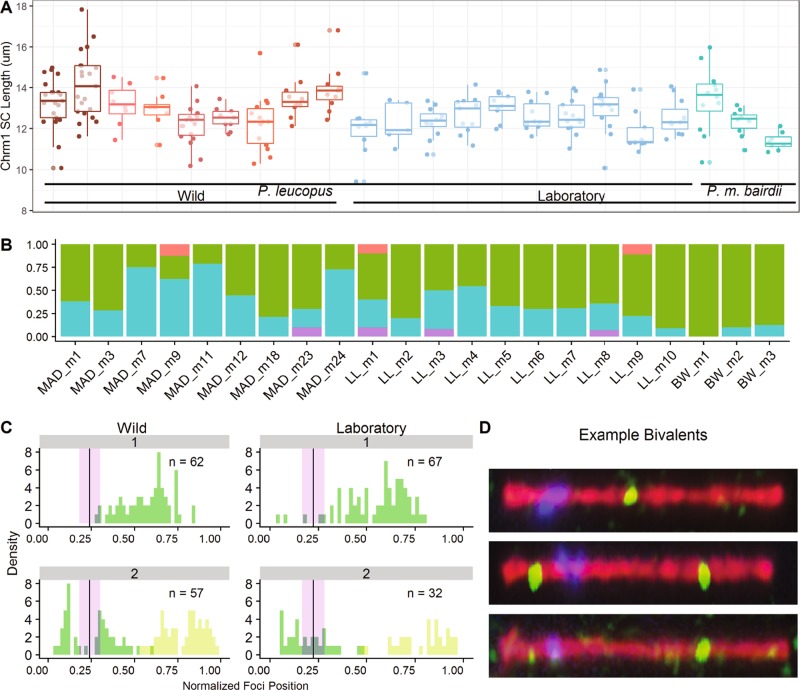
**a** Distributions of synaptonemal complex length for chromosome 1. Colors match those in Fig. 1. Each point represents a chromosome 1 SC length for a single spermatocyte. Boxplots overlaid to illustrate mouse means. **b** Barplots of proportions of chromosome 1 observations with 0 (pink), 1 (green), 2 (blue) or 3 (purple) crossovers. The ordering of Mouse IDs corresponds to that in **a**. **c** Positions of MLH1 foci normalized by synaptonemal complex length. Observations are placed in 5% bins and divided into classes with one or two MLH1 foci. Number of spermatocytes surveyed is listed in upper right corner. Dark green = first focus in proximity to the centromere; light green = second focus in proximity to the centromere. The mean centromere position is indicated by the vertical line at 0.25, with purple shading indicating ± 2 S.E. **d** Representatives of automated output for segmented chromosome 1 bivalents with 1, 2, and 3 foci

Isolation of single bivalents is based primarily on red signal and the elongated shape of pachytene stage bivalents. SC length is quantified as the straightened midline. Locations of the centromere and MLH1 foci are quantified along the length of this midline and reported in a .csv file. Two types of image files are returned for each image to match measures with objects in the original image and to quickly assess the algorithm’s performance in isolating single bivalents. The “whole.tif” file displays a box around all identified objects and an index at objects that pass heuristic criteria (Fig. 1c). The second file type, with the suffix “straight2.tif”, displays a cropped portion of the original image and a representation of the features detected by the algorithm (Fig. 2c). Additional examples of output files are included in Supplemental Data File S1.

To quantify the accuracy of DNACrossover, we generated a dataset of hand measured bivalents from eight wild *P. leucopus* spermatocytes (taken from seven mice), two laboratory-raised *P. leucopus* spermatocytes (from two mice), and two *P. m. bairdii* spermatocytes (from two mice). SC length, centromere position, and MLH1 foci position (ordered by proximity to centromere) were measured and matched to the corresponding automated measure on the same bivalent.

**Fig. 3:**
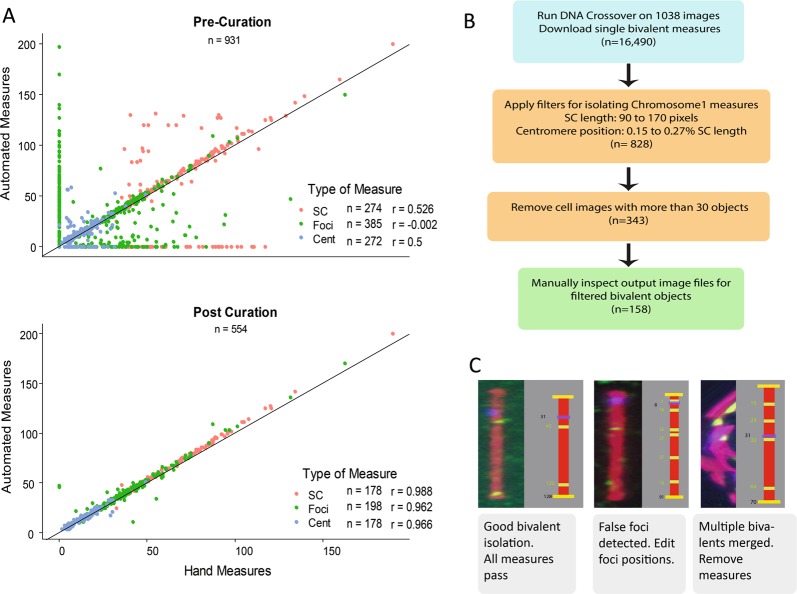
**a** Comparison of automated to hand measures, separated by Pre- and Post-curation steps. The total number of observations and Pearson’s *r* values are listed in the legend. **b** Steps for isolation of chromosome 1 measures from the automated single bivalent dataset. Boxes are color coded by process: white: CyVerse-DNA crossover method, beige: custom R scripts, green: manual curation. **c** Example of single bivalent output files and the logic applied during the curation step

To characterize SC length and crossover position from spermatocyte images, we focused on chromosome 1, which could be uniquely identified as the metacentric chromosome with the longest SC in both *Peromyscus* species (Mlynarski et al. 2008). In comparisons of chromosome 1 SC length across cells and mice, we assumed that physical chromosome size (in base pairs) was constant, with different SC lengths reflecting variation of the chromatin loop size and chromosome axis length (i.e. packing ratio) (Kleckner et al. 2003). All chromosome 1 bivalents were manually verified for correct segmentation before being included in downstream analyses. The accuracy of the pipeline was assessed by comparing automated results with measurements made manually using Fiji (ImageJ v1.52) (Schindelin 2012). The relationship between automated and manual measurements was summarized using the Pearson’s correlation coefficient. SC length, centromere position, and MLH1 foci position were considered separately (Fig. 3). Initial analysis of SC lengths was done in pixels and subsequently converted to micrometers with a translation factor of 9.8152 pixels per micrometer.

### Statistical analyses

Statistical analyses were performed in R (v3.5.2) (RStudio Team, 2015) using custom scripts. We used the lmer4 package (Bates et al. 2015) to build sets of mixed models that evaluated the effects of mouse, sampling location, and month (for wild mice), wild vs laboratory origin, species, and image/cell quality on MLH1 count and SC length. Mixed models were chosen so that the factor of mouse ID could be coded as a random effect to partition variances between individuals and to mitigate pseudo-replication. We used likelihood ratio tests (LRTs) to evaluate the significance of estimated coefficients, within the drop1 from lme4 and exactRLRT from the RLRsim package (Scheipl et al. 2008) functions to applied fixed and random effects respectively. Since the lowest level, mouse ID, was uniquely coded in the dataset, nesting was implicit and not explicitly coded in the analysis. The number of MLH1 foci in a spermatocyte took on an integer value, complicating model-based analyses. We used two approaches to mitigate this issue in our analyses of mixed models. First, we inspected histograms of MLH1 focus count. When the count was approximately normal, we proceeded with mixed models using the count in each spermatocyte as the dependent variable. Second, we build mixed models with the square root of count data to stabilize the variance.

Levels of variation in MLH1 count within wild mice and within laboratory-raised mice were compared using two permutation tests. To test whether the average within-mouse variance in MLH1 count differed, the status label (wild/laboratory) of within-mouse variance in MLH1 count was randomized. To test whether levels of variation in MLH1 count differed at the group level, the status label for observations of mouse mean MLH1 count was randomized. A similar approach was applied to chromosome 1 SC length statistics and interference strength.

To evaluate the ability of chromosome 1 SC length to predict whether the bivalent harbored 1 or 2 MLH1 foci, we used logistic regression. Normalized foci positions (position/SC length) were compared by categorizing chromosome 1 bivalents into single- and double-focus (crossover) classes, grouping into 5% bins, and applying a Kolmogorov–Smirnov test. In addition to our comparison of the normalized foci positions, we calculated the intra-chromosomal portion of $\bar r$ (Veller et al. 2019) to compare how evenly crossovers were spaced while accounting for raw SC length. This metric ranges from 0 to 0.5, with higher values indicating that SC proportions flanking crossovers are more equivalent.

To evaluate the strength of crossover interference, we fit the distance between MLH1 foci (on chromosome 1 bivalents with two foci; pooled across mice) to a gamma distribution and estimated the shape parameter, ν (“nu”) (McPeek and Speed 1995; Broman et al. 2002; de Boer et al. 2006). We used the egamma function from the EnvStats package (Millard 2013). We considered absolute distance and normalized distance (absolute distance/SC length) in separate analyses.

## Results

### Little variation in recombination rate among wild *P. leucopus*

The genome-wide recombination rate was manually quantified by counting MLH1 foci along bivalents in each of 221 spermatocytes from a total of nine wild male *P. leucopus* (Table 1, Fig. 1). We observed a global average across mice of 23.5 MLH1 foci per cell, with individual mouse means ranging from 22.92 to 24.53 (Table 1). These results indicated that the recombination rate in wild *P. leucopus* is low and near a threshold of one crossover per bivalent thought to be necessary to avoid chromosomal non-disjunction. Sixteen percent of pooled spermatocytes had fewer than 23 MLH1 foci and 3% of all bivalents scored in wild mice lacked MLH1 foci. Although some of these bivalents could have been achiasmate, some might have harbored crossovers generated by the MLH1-independent pathway, which is estimated to produce up to 10% of crossovers in house mice (Holloway et al. 2008).

**Table 1:** MLH1 count statistics for single mice

Species	Status	Mouse ID	No. of cells	Mean	SD	SE	CV
*P. leucopus*	Wild	MAD_m1	26	23.269	0.667	0.131	2.865
		MAD_m3	28	23.393	0.832	0.157	3.555
		MAD_m6	12	22.917	1.084	0.313	4.728
		MAD_m7	19	23.474	1.219	0.28	5.192
		MAD_m18	37	23	1.179	0.194	5.124
		MAD_m11	30	24.533	1.279	0.234	5.215
		MAD_m12	28	23.607	0.956	0.181	4.05
		MAD_m23	15	23.533	1.302	0.336	5.533
		MAD_m24	26	23.808	1.877	0.368	7.882
		**Group**	**221**	**23.53**	**1.3**	**0.085**	**5.4**
*P. leucopus*	Lab-raised	LL_m1	33	22.909	1.487	0.259	6.489
		LL_m3	29	22.552	1.594	0.296	7.07
		LL_m4	23	22.217	1.93	0.402	8.685
		LL_m5	22	23	1.69	0.36	7.349
		LL_m6	22	23.318	0.839	0.179	3.597
		LL_m7	29	21.862	1.382	0.257	6.32
		LL_m8	28	22.107	1.685	0.318	7.623
		LL_m9	28	23.107	1.397	0.264	6.045
		LL_m10	29	22.552	1.682	0.312	7.456
		**Group**	**243**	**22.61**	**1.521**	**0.294**	**7.033**
*P. m. bairdii*	Lab-raised	BW_m1	22	23	1.543	0.329	6.709
		BW_m2	31	22.774	1.146	0.206	5.033
		**Group**	**53**	**22.87**	**1.316**	**0.181**	**5.755**

The mean MLH1 count, standard deviation (*SD*), standard error (*SE*), and coefficient of variation (*CV*) are calculated at the mouse and group level (bold)

Inspections of histograms revealed that distributions of MLH1 foci counts across spermatocytes were approximately normal (Supplemental Fig. 1). In a mixed model treating MLH1 count as the dependent variable, mouse (as a random effect) and cell quality (as a fixed effect) were significant factors (*p* < 0.05) (Tables 2 and 3).

**Table 2:** Four mixed models for MLH1 counts and results of likelihood ratio test (LRT) for fixed and random effects

**Laboratory-raised** ***P. leucopus and P. m. bairdii*** **MLH1 counts**				
**Fixed effects**	**Effect size**	**SE**	**LRT**	***p*** **value**
Intercept	23.570	0.325		
Species	0.250	0.378	0.52	0.47017
Quality	−0.320	0.094	11.13	0.00085
**Random effects**	**Variance**	**SD**	**LRT**	***p*** **value**
Mouse	0.150	0.388	6.32	0.0039
Residual	2.196	1.482		
**All** ***P. leucopus*** **MLH1 counts**				
**Fixed effects**	**Effect size**	**SE**	**LRT**	***p*** **value**
Intercept	23.601	0.258		
Status	0.700	0.240	7.64	0.0057
Quality	−0.328	0.066	23.97	9.80 e–07
**Random effects**	**Variance**	**SD**	**LRT**	***p*** **value**
Mouse	0.178	0.421	20	<2e-16
Residual	1.836	1.355		
**Wild** ***P. leucopus*** **MLH1 counts**				
**Fixed effects**	**Effect size**	**SE**	**LRT**	***p*** **value**
Intercept	22.203	1.733		
Month	0.241	0.241	0	–
Feb.	0.670	0.410		
July	0.345	0.420		
June	−0.048	0.480		
Location 1	−0.170	0.415	0.450	0.5
Quality	−0.312	0.078	15.88	6.70e-05
**Random effects**	**Variance**	**SD**	**LRT**	***p*** **value**
Mouse	0.099	0.314	2.87	0.027
Residual	1.338	1.157		
**Laboratory-raised** ***P. leucopus*** **MLH1 counts**				
**Fixed effect**	**Effect size**	**SE**	**LRT**	***p*** **value**
Intercept	23.682	0.360		
Quality	−0.356	0.108	10.8	0.001
**Random effect**	**Variance**	**SD**	**LRT**	***p*** **value**
Mouse	0.155	0.394	5.54	0.0058
Residual	2.298	1.516

**Table 3:** Four mixed models for square root of MLH1 counts and results of likelihood ratio test (LRT) for fixed and random effects

**Laboratory-raised** ***P. leucopus and P. m. bairdii*** **square root MLH1 counts**				
**Fixed effect**	**Effect size**	**SE**	**LRT**	***p*** **value**
Intercept	4.855	0.034		
Species	0.027	0.040	0.55	0.45942
Quality	−0.034	0.010	11.46	0.00071
**Random effect**	**Variance**	**SD**	**LRT**	***p*** **value**
Mouse	0.002	0.041	6.44	0.0033
Residual	0.024	0.156		
**All** ***P. leucopus*** **square root MLH1 counts**				
**Fixed effect**	**Effect size**	**SE**	**LRT**	***p*** **value**
Intercept	4.857	0.027		
Status	0.074	0.025	7.79	0.0053
Quality	−0.035	0.007	24.45	7.60 e–07
**Random effect**	**Variance**	**SD**	**LRT**	***p*** **value**
Mouse	0.002	0.044	19.4	2.00 e–16
Residual	0.020	0.142		
**Wild** ***P. leucopus*** **square root MLH1 counts**				
**Fixed effect**	**Effect size**	**SE**	**LRT**	***p*** **value**
Intercept	4.913	0.039		
Month			0	
Feb.	0.069	0.042		
July	0.034	0.043		
June	−0.051	0.049		
Location 1	−0.017	0.042	0.46	
Quality	−0.033	0.008	16.53	4.80 e–05
**Random effect**	**Variance**	**SD**	**LRT**	***p*** **value**
Mouse	0.001	0.031	3	0.03
Residual	0.014	0.119		
**Laboratory-raised** ***P. leucopus*** **square root MLH1** **counts**				
**Fixed effect**	**Effect size**	**SE**	**LRT**	***p*** **value**
Intercept	4.866	0.038		
Quality	−0.038	0.011	10.9	0.00094
**Random effect**	**Variance**	**SD**	**LRT**	***p*** **value**
Mouse	0.002	0.042	5.64	0.0064
Residual	0.026	0.160		

### Similar levels of recombination rate variation in wild and laboratory-raised *P. leucopus*

The average MLH1 count across 243 spermatocytes taken from a total of nine laboratory-raised *P. leucopus* was 22.62 foci per cell. This value was 0.8 foci lower than the average in wild *P. leucopus*, a small but significant reduction in recombination rate (*t*-test; *p* = 0.0015). This difference between laboratory-raised and wild *P. leucopus* persisted in mixed models treating either raw MLH1 counts or square-root MLH1 counts as dependent variables (mixed model LRT; *p* = 0.0057 and *p* = 0.0053, respectively) (Tables 2 and 3). Five percent of bivalents across cells lacked MLH1 foci and 45% of pooled spermatocytes had fewer than 23 MLH1 foci.

We found little variation in average MLH1 counts among laboratory-raised mice, with mouse averages ranging between 21.86 and 23.32 foci per cell. Permutation tests revealed that wild and laboratory-raised *P. leucopus* samples did not differ in among-mouse variance in MLH1 count (*p* = 0.52; 10,000 permutations). In contrast, laboratory-raised *P. leucopus* had higher within-mouse variance in MLH1 count than wild *P. leucopus* (*p* = 0.0011; 10,000 permutations).

### Conservation of recombination rate between laboratory-raised *P. leucopus* and *P. maniculatus bairdii*

The average MLH1 count across 53 spermatocytes from two *P. m. bairdii* was 22.61 foci per cell. There was no evidence for a difference in MLH1 count between *P. m. bairdii* and *P. leucopus* raised in the same colony (*t*-test; *p* = 0.24) (mixed model LRT; *p* = 0.47; Tables 2 and 3). MLH1 count was lower in *P. m. bairdii* compared to wild *P. leucopus* (*t*-test; *p* = 0.0013). Three percent of *P. m. bairdii* bivalents lacked MLH1 foci and 34% of pooled spermatocytes had fewer than 23 MLH1 foci.

MLH1 count averages for several individuals from laboratory-raised *P. leucopus* and *P. m. bairdii* fell below 23, the expectation based on one crossover per chromosome. Although this pattern suggests that recombination rates in these mice are close to the predicted lower bound, there are reasons to suspect that achiasmy is not the primary explanation, including the possibility of compensatory type II crossovers.

### Accurate measurement of synaptonemal complex length using automated method

In our dataset of spermatocyte images, an average of 21.5 chromosome objects was identified per cell (compared to the expectation of 24 chromosomes). An average of 16.28 identified chromosome objects met the bivalent criteria; their measurements were reported by the algorithm. Even in cells with a good spread, a portion of overlapping bivalents could not be isolated by the algorithm. A weakness with the algorithm is that multiple overlapping bivalents may be merged into one bivalent, leaving a small proportion of single bivalents unmeasured as indicated by chromosome shapes without boxes or indices (Fig. 1c).

We measured the accuracy of automated estimates of SC length, MLH1 foci position, and centromere position by comparing them to manual estimates. We compared automated and manual estimates across two datasets: a pre-curation dataset (in which automated estimates were unfiltered) and a post-curation dataset (in which errors were diagnosed based on output files and removed) (Fig. 2a). Automated estimates using the pre-curation dataset showed low correlations to manual estimates (Pearson’s *r* = 0.52, −0.002, and 0.5 for SC length, MLH1 foci position, and centromere position, respectively). Primary sources of error were extra foci (foci detected automatically but not manually) and incorrectly merged bivalents. Diagnosis of these errors was straightforward, as illustrated in Fig. 2 and Supplemental File 1. The removal of 60% of observations in the construction of the post-curation dataset resulted in very high correlations between automated and manual estimates (Pearson’s *r* = 0.966, 0.964, 0.981, for SC length, MLH1 foci position, and centromere position, respectively) (Fig. 2). In the post-curation dataset, automated estimates of SC length were between 3% and 5% greater than manual estimates, a difference on the same scale as error introduced by multiple human measurers.

We applied a series of criteria to the automated data set to isolate 158 chromosome 1 observations based on SC length and centromere position (Fig. 2b). We manually verified that each putative chromosome 1 observation came from the longest bivalent in the cell and that MLH1 foci positions were correct. Initial bivalent identification by the algorithm can be affected by the quality of the cell spread; the algorithm failed to produce automated estimates from some mice with tightly packed cells. Even with the shortcomings of our algorithm, its ability to rapidly process hundreds of single cell images and produce accurate estimates for a curated subset offers a substantial time savings compared to manual measurement. In our experience, manually measuring bivalents from a single cell image took approximately 30 min, whereas our DNACrossover tool processed our dataset of >700 images in approximately 8 hours.

### Variation in chromosome 1 synaptonemal complex length

One-hundred fifty-eight automated measurements of chromosome 1 SC length were supplemented with 97 manual measurements to ensure that all mice had close to 10 observations (Fig. 3, Table 4). The average manual and automated SC lengths for laboratory-raised *P. leucopus* were 12.53 and 12.52 μm, respectively, while the averages for wild *P. leucopus* were 13.11 and 13.14 μm for manual and automated measures. We observed no difference between the supplemented manual and automated measures, (*t*-test; *p* = 0.9, *p* = 0.2, for laboratory and wild observations, respectively). Since SC staining was more robust than MLH1 staining, some SC observations were included from mice that did not have sufficient MLH1 signal to be included in the first analysis.

**Table 4:** SC length statistics for chromosomes 1 listed for mouse and group level (bold)

Species	Status	Animal ID	No. of bivalents	Mean (µm)	SD	SE	No. 1 focus (%)	No. 2 foci (%)	Mean $\bar r$
*P. leucopus*	Wild	MAD_m1	21	13.264	1.218	0.266	13 (62%)	8 (38%)	0.239
		MAD_m3	21	14.137	1.608	0.351	15 (71%)	6 (29%)	0.228
		MAD_m7	8	13.174	1.017	0.36	2 (25%)	6 (75%)	0.248
		MAD_m9	8	12.939	0.929	0.328	2 (25%)^a^	5 (63%)^a^	0.243
		MAD_m11	19	12.194	0.919	0.211	4 (21%)	15 (79%)	0.268
		MAD_m12	9	12.486	0.554	0.185	5 (56%)	4 (44%)	0.259
		MAD_m18	14	12.291	1.379	0.369	11 (79%)	3 (21%)	0.232
		MAD_m23	10	13.520	1.093	0.346	7 (70%)^b^	2 (20%)^b^	0.249
		MAD_m24	11	13.921	1.142	0.344	3 (27%)	8 (73%)	0.272
		**Group**	**121**	**13.103**	**1.400**	**0.120**	**62 (51%)**	**57 (47%)**	**0.247**
*P. leucopus*	Lab-raised	LL_m1	10	12.022	1.329	0.42	5 (50%)^a,b^	3 (30%)^a,b^	0.221
		LL_m2	5	12.256	1.03	0.461	4 (80%)	1 (20%)	0.233
		LL_m3	12	12.300	0.735	0.212	6 (50%)^b^	5 (42%)^b^	0.256
		LL_m4	11	12.682	0.896	0.27	5 (45%)	6 (55%)	0.244
		LL_m5	9	13.060	0.650	0.194	6 (66%)	3 (33%)	0.241
		LL_m6	10	12.628	0.756	0.239	7 (70%)	3 (30%)	0.242
		LL_m7	13	12.583	0.83	0.23	9 (69%)	4 (31%)	0.214
		LL_m8	14	13.009	1.162	0.311	9 (64%)^b^	4 (29%)^b^	0.220
		LL_m9	9	11.819	0.966	0.322	6 (67%)^a^	2 (22%)^a^	0.223
		LL_m10	11	12.546	0.861	0.26	10 (91%)	1 (9%)	0.207
		**Group**	**104**	**12.490**	**0.973**	**0.095**	**67 (64%)**	**32 (32%)**	**0.230**
*P. m. bairdii*	Lab-raised	BW_m1	12	13.446	1.571	0.454	12 (100%)	0 (0)	0.205
		BW_m2	10	12.308	0.652	0.206	9 (90%)	1 (10%)	0.210
		BW_m3	8	11.375	0.428	0.139	7 (87%)	1 (12%)	0.233
		**Group**	**30**	**12.376**	**1.360**	**0.240**	**28 (93%)**	**2 (6%)**	**0.215**

Species, status, mouse ID, and number of chromosome 1 observation are listed for each mouse and pooled for groups. The mean SC length (in μm), standard deviation (*SD*), and standard error (*SE*) are calculated. Numbers of 1-focus and 2-foci observations and their percentages are listed. Percentages that do not add to 100 are due to either single instances with 0-focus or 3-foci bivalents, marked by “a” or “b” respectively. Mean intra-chromosomal $\bar r$ for all mouse or group level observations is calculated.

Wild *P. leucopus* caught during different months and/or from different locations had different SC lengths (month and location were confounded; Fig. 3; Table 4). Average SC length (for a wild mouse) ranged from 12.19 to 14.14 μm, with weak evidence for statistical differences among wild mice indicated by the mixed model (mixed model LRT; *p* = 0.048) (Table 5). SCs were longer in wild *P. leucopus* than in laboratory-raised *P. leucopus* (*t*-test; *p* = 1.3e-4). There was no evidence that SC length differed between *P. leucopus* and *P. m. bairdii* raised in the same laboratory (*t*-test: *p* = 0.21). The amount of between-mouse variation in the wild was higher for SC length than for MLH1 count. Permutation tests revealed that SC length varied more among wild *P. leucopus* than among laboratory-raised *P. leucopus* (*p* = 4e-4; 10,000 permutations). Within mouse variance was also greater in wild mice (*p* = 0.020; 10,000 permutations).

**Table 5:** Four mixed models for SC lengths and results of likelihood ratio test (LRT) for fixed and random effects

**Laboratory-raised** ***P. leucopus and P. m. bairdii*** **chromosome 1 SC length**				
**Fixed effect**	**Effect size**	**SE**	**LRT**	***p*** **value**
Intercept	116.699	2.792		
Species	0.345	3.723	0	0.9792
Number of MLH1 Foci	4.491	1.614	7.62	0.0058
**Random effect**	**Variance**	**SD**	**LRT**	***p*** **value**
Mouse	22.720	4.766	14	1.00e-04
Residual	87.100	9.333		
**All** ***P. leucopus*** **chromosome 1 SC length**				
**Fixed effect**	**Effect size**	**SE**	**LRT**	***p*** **value**
Intercept	116.926	2.581		
Status	5.337	2.707	3.86	0.0496
Number of MLH1 Foci	4.326	1.32	10.41	0.0013
**Random effect**	**Variance**	**SD**	**LRT**	***p*** **value**
Mouse	24.660	4.966	28	<2e-16
Residual	107.890	10.387		
**Wild** ***P. leucopus*** **chromosome 1 SC length**				
**Fixed effect**	**Effect size**	**SE**	**LRT**	***p*** **value**
Intercept	121.856	6.537		
Number of MLH1 Foci	4.255	2.117	3.68	0.055
Month			0	
Feb.	−7.979	6.353		
July	6.513	6.456		
June	−6.379	7.223		
Oct.	−1.238	7.635		
Location 1	6.947	6.256	2.08	0.15
**Random effect**	**Variance**	**SD**	**LRT**	***p*** **value**
Mouse	12.360	3.516	2.120	0.048
Residual	133.170	11.540		
**Laboratory-raised** ***P. leucopus*** **chromosome 1 SC length**				
**Fixed effect**	**Effect size**	**SE**	**LRT**	***p*** **value**
Intercept	116.712	2.417		
Number of MLH1 Foci	4.533	1.569	8.04	0.0046
**Random effect**	**Variance**	**SD**	**LRT**	***p*** **value**
Mouse	6.379	2.526	2.16	0.053
Residual	79.579	8.921		

### Synaptonemal complex length, crossover number, and crossover position

For analyses of MLH1 foci position along chromosome 1, we focused on *P. leucopus* samples and we pooled observations to increase power. In the majority of mixed models treating SC length as the dependent variable, mouse (as a random effect) and number of foci (as a fixed effect) were significant factors (Table 5). Only in the mixed model of wild *P. leucopus* SC length as a dependent variable the number of foci per bivalent was a non-significant factor (LRT; *p* = 0.055) (Table 5). A small number of chromosome 1 bivalents had 0 foci (0.8%) or 3 foci (0.8%) (Fig. 3b). To simplify analyses of foci position and relationships between foci number and SC length, these bivalents were excluded. Logistic regression revealed that longer chromosome 1 SCs were more likely to harbor 2 foci instead of 1 focus for *P. leucopus* raised in the laboratory (*p* = 1.69e-3). For wild *P. leucopus*, the logistic regression for this relationship was not significant (*p* = 0.46) unless month (as an integer) was added as a variable (*p* = 0.095, *p* = 0.002 for SC length and month, respectively). Closer examination of SC lengths showed that within two wild mice, a subset of bivalents with one focus had longer SC lengths than the average for two-foci bivalents (Supplemental Fig. 2).

*P. leucopus* showed strong, positive interference between crossovers along chromosome 1 in both wild (*ν*_absolute_ = 11.1; *ν*_normalized_ = 14.09; *n* = 37 bivalents) and laboratory (*ν*_absolute_ = 10.0; *ν*_normalized_ = 9.34; *n* = 27 bivalents) samples (a single bivalent with an outlying inter-focal distance of 0.82 μm was removed prior to conducting these analyses). Bivalents with 1 vs. 2 foci showed distinct patterns in crossover position. Foci on single-focus bivalents were localized near the center of the SC, whereas foci on double-focus bivalents were displaced toward the ends of the SC (Fig. 3)—another sign of interference. The distributions of normalized foci positions were similar in wild and laboratory-raised *P. leucopus* (two-sample Kolmogorov–Smirnov; *p* = 0.98 for single-focus bivalents; *p* = 0.49 for double-focus bivalents).

The average intra-chromosomal $\bar r$ values for chromosome 1 bivalent classes from *P. m. bairdii* are (0.21 and 0.22 for single- and double-focus bivalents, respectively), laboratory-raised *P. leucopus* (0.22 and 0.26), and wild *P. leucopus* (0.23 and 0.27). We observed no significant differences in intra-chromosomal $\bar r$ values between *P. leucopus* and *P. m. bairdii* (*t*-test; *p* = 0.4 and *p* = 0.7, for single-focus and double-focus bivalents, respectively) nor between wild and laboratory-raised *P. leucopus* (*t*-test; *p* = 0.3 and *p* = 0.2, for single-focus and double-focus bivalents, respectively). When the intra-chromosomal $\bar r$ values were compared within group and across bivalent class (single-focus to double-focus bivalents), they were significantly different (*t*-test; *p* = 2e-09 and *p* = 0.003 for wild and laboratory-raised *P. leucopus* respectively).

We found a total of four chromosome 1 bivalents with three foci (1.6% of surveyed bivalents) (Fig. 3b). This low number of observations prevented us from analyzing the positions of these foci within a statistical framework, but we noted that the foci had size and brightness similar to the distinct positions of single-focus and double-focus bivalents (Fig. 3d). SCs for these 3-foci bivalents were not significantly longer than SCs for bivalents with one or two foci within the same mouse (Supplemental Fig. 2).

## Discussion

### Low variation in the genome-wide recombination rate in white-footed mice

Our estimates of the genome-wide recombination rate from *P. leucopus* and *P. m. bairdii* are similar to previous characterizations of recombination in *Peromyscus*. Assuming one MLH1 focus is equivalent to 50 cM, we estimate the average autosomal genetic map length for wild *P. leucopus* to be 1175.2 cM, approximately 87% of the estimate (1349 cM) from an inter-specific backcross between *P. maniculatus* × *P. polionotus* (Kenney-Hunt et al. 2014). This difference is modest, considering that genetic maps detect the approximately 10% of type II (non-interfering) crossovers that do not involve MLH1 (Holloway et al. 2008). Additionally, our estimates resemble spermatocyte MLH1 counts from a small number of *P. leucopus* previously sampled in Madison (Mann–Whitney *U* test; *p* = 0.4) (Dumont and Payseur 2011b).

MLH1 count averages for several individuals from laboratory-raised *P. leucopus* and *P. m. bairdii* fell below 23, the expectation based on one crossover per chromosome. Forty-five percent and 34% of all bivalents lacked MLH1 foci in *P. leucopus* and *P. m. bairdii*, respectively. Interpretation of this cell-to-cell variation would require an experimental design that can distinguish whether these mice have compensatory type II crossovers or elevated rates of achiasmy. Recent work documented covariation in crossover number among bivalents within nuclei (Wang et al. 2019). One consequence of this pattern is that the number of crossovers per cell is over-dispersed, suggesting that one crossover per chromosome may not be a strict rule for all cells within an individual. The fitness effects of the variation in crossover counts within an individual still needs further investigation.

Quantifying variation in recombination rate among individuals from a wild population was the primary goal of our study. To help interpret observed patterns, we compared wild and laboratory-raised populations. Although we found inter-individual rate variation in wild mice, the level of variation was low and similar to that seen among mice raised in the laboratory. This observation seems surprising on theoretical grounds. We expect a wild population to have a higher effective population size than a closed laboratory colony that recently underwent a strong contraction (see PSC website), and quantitative genetic models predict that the standing level of genetic variation in a trait will scale with effective population size (Walsh and Lynch 2018). Moreover, mice likely experience greater environmental heterogeneity in the wild than in the laboratory.

Multiple environmental factors—including temperature (Hotta et al. 1988; Bomblies et al. 2015) and parasites (Singh et al. 2015; Jackson et al. 2015)—are known to affect recombination rate. Although most such findings came from exotherms (plants and invertebrates) (Modliszewski and Copenhaver 2017; Morgan et al. 2017), a number of experiments in house mice documented effects of temperature and BPA exposure on the recombination rate (Hotta et al. 1988; Susiarjo et al. 2007; Vrooman and Hunt 2011), again suggesting that rate variation should be higher in wild mice. While our experimental design was not suited to examine environmental or developmental variables, neither a wild mouse infected with a large botfly larva (MAD_m1) nor a wild mouse that was a juvenile (MAD_m3) exhibited different recombination rates from the remainder of the sample (*t*-test; *p* = 0.38, *p* = 0.068, for MAD_m3 and MAD_m1, respectively).

We propose three interpretations to explain the low levels of recombination rate variation in nature and in the laboratory. First, the wild population could have contracted recently, making the effective population sizes of the two groups more similar. An estimate of nucleotide diversity at a mitochondrial locus in our wild sample of *P. leucopus* (*π* = 0.004) is lower than an estimate from a sample of a Canadian population of *P. leucopus* using the same primers (*π* = 0.017) (Fiset et al. 2015). This difference is in line with the notion that the local Madison population of *P. leucopus* underwent a bottleneck. If a recent bottleneck occurred, theory would predict that selection on unrelated traits could indirectly generate selection for elevated recombination rate to minimize Hill–Robertson interference (Otto and Barton 2001). However, the difference in mitochondrial diversity could also reflect the smaller geographic range over which we sampled. Another possibility is that reduced variation at mitochondrial loci is not representative of patterns in most of the genome, where there could be less evidence for a population contraction.

A second potential explanation for the low level of variation in recombination rate we observed is that the mutational target size for this trait is relatively small in wild populations. Two patterns seem to contradict this explanation. Molecular genetic studies indicate that tens to hundreds of genes are involved in determining the recombination rate (Hunter 2015). Furthermore, standing differences in recombination rate among strains of house mice are polygenic (Murdoch et al. 2010; Dumont and Payseur 2011a). On the other hand, a common set of genes correlates with individual differences in recombination rate in multiple mammalian species (Kong et al. 2008, 2014; Chowdhury et al. 2009; Sandor et al. 2012; Ma et al. 2015; Johnston et al. 2016).

The third and more plausible explanation for our findings is that stabilizing selection reduces variation in the genome-wide recombination rate—in nature and in the laboratory—by targeting recombination or a correlated trait. Although there are few other empirical studies of variation in the genome-wide recombination rate in wild populations, MLH1 counts from multiple wild-derived inbred strains revealed a low amount of variation within subspecies of house mice (Dumont and Payseur 2011b). Stabilizing selection could also explain the similarity in genome-wide recombination rates between species of New World murids (24.71, 26.49, 23.74, 23.7, 22.87, and 22.06 crossovers per cell for *M. ochrogaster, M. mogollonensis*, *M. pennsylvanicus*, *P. leucopus, P. maniculatus*, and *P. polionotus*, respectively; Dumont and Payseur, 2011b; Kenney-Hunt et al. 2014; Dumont 2017). Inferred increases in the genome-wide recombination rate across mammalian species (consistent with directional selection; Segura et al. 2013) further suggest the existence of distinct optima for different species groups.

The notion that crossover counts are subject to stabilizing selection should motivate research to better characterize cellular limits on the genome-wide recombination rate (Ritz et al. 2017). Most sexual species require a minimum of one crossover per chromosome for proper segregation during meiosis (Hassold and Hunt 2001; Coop and Przeworski 2007; Fledel-Alon et al. 2009; Dumont 2017), indicating a possibly strong lower bound. In contrast, upper limits on the genome-wide recombination rate remain poorly understood, though some potentially relevant factors have been identified (Hassold and Hunt 2001; Inoue and Lupski 2002; Ritz et al. 2017). Testing the hypothesis that the genome-wide recombination rate experiences stabilizing selection will require measuring the connection between this trait and fitness. We also note that alternative evolutionary models could explain variation in the recombination rate on finer genomic scales not captured in this study (e.g. recombination hotspots; Smukowski and Noor 2011; Dapper and Payseur 2017).

### Crossover patterning along a single chromosome in white-footed mice

Since SC length and crossover interference can influence the genome-wide recombination rate by modulating crossover number and placement on single chromosomes, we quantified SC length and interference strength for chromosome 1. Observed patterns from these single chromosome measures confirm that physical distances along pachytene bivalents provide valuable metrics for characterizing features of meiosis (Zhang et al. 2014). Assuming that all chromosome 1 observations have the same physical amount of DNA across cells, the distribution of chromosome 1 SC length within a mouse reflects differences in the packing ratios across cells.

Our inferences about SC length and crossover interference were substantially accelerated by the automated image analysis software we developed. Estimates produced by this approach were similar to manual estimates, and our method could be applied in a high-throughput fashion with minimal manual curation. We see the software as a helpful tool that could be used to characterize crossover patterning in new and existing immunofluorescent datasets in other species (Zhang et al. 2014).

In contrast to the genome-wide recombination rate, SC length variation was higher in wild mice than in laboratory-raised mice. We see two potential contributors to this pattern. First, slides from wild mice were prepared on different days, whereas slides from laboratory-raised mice were prepared on the same day. Variable laboratory conditions that could affect spermatocyte spreads (e.g. humidity) might have increased the level of technical error in wild mice. Second, the higher variation in SC length in wild mice could reflect seasonal effects of the natural environment (e.g. temperature). Although month was not a significant factor in the mixed model of SC length in wild mice, we noticed a symmetrical pattern in SC length across months (Supplemental Fig. 3). Additional work will be needed to distinguish these possibilities. We did not find evidence for a seasonal effect on MLH1 count.

Our results indicate that crossover number and placement are closely tied in *P. leucopus*. There are two complementary classes of bivalents for chromosome 1: those with a single MLH1 focus near the middle and those with two foci at the ends (Fig. 3). This pattern is consistent with a model in which the interference distance between crossovers is slightly less than the length of the chromosome. A similar distinction in crossover positions has been reported for large chromosomes in house mouse spermatocytes (Froenicke et al. 2002) and in other species (e.g. Basheva et al. 2008, 2010; Davenport et al. 2018). The difference in intra-chromosomal $\bar r$ values between bivalent classes and the lack of difference across status and species we observed aligns with these findings.

Our inference of strong, positive interference from the distances between pairs of MLH1 foci provides another sign that crossover number and placement are related. Our interference estimates (*ν*_absolute_ = 11.1; *ν*_normalized_ = 14.09, wild mice; *ν*_absolute_ = 10.0; *ν*_normalized_ = 9.34; laboratory-raised mice) are consistent with published estimates for laboratory house mice generated from linkage (*ν* = 11.3; Broman et al. 2002) and cytological (*ν* = 13.7; de Boer et al. 2006) data. Overall, the similarity of crossover positions and interference strength between wild and laboratory-raised *P. leucopus* suggest that the regulation of crossover patterning is conserved, at least at a broad chromosomal scale.

One observation that is more difficult to explain is that the percentages of chromosome 1 bivalents with either zero or three MLH1 foci were higher in laboratory-raised *P. leucopus* (zero foci: 1.9%; three foci: 2.8%) than wild *P. leucopus* (zero foci: 0.8%; three foci: 0.8%). With the MLH1 approach alone, we cannot determine whether bivalents lacking MLH1 foci are truly achiasmate; some of these bivalents could harbor type II crossovers and others could represent technical errors, including issues with antibody specificity. In addition, some of these bivalents could have been sampled outside the temporal window during which MLH1 foci are detectable. Bivalents with three foci did not have significantly longer SC lengths (Supplemental Fig. 2). The spatial distribution and foci morphology on three-foci bivalents could be due to interactions at earlier stages when interference acted on multiple crossover positions (de Boer et al. 2006; Zhang et al. 2014), though the specifics of such interactions are poorly understood and it is hard to see why this phenomenon would differ between wild and laboratory-raised mice.

Our interpretations are accompanied by a few additional caveats. As mentioned previously, our interpretations are restricted to type I (interfering) crossovers. If numbers of type II (non-interfering) crossovers vary between groups our inference of conservation in the genome-wide recombination rates would be less accurate. Crossover number, SC length, and crossover patterning are sexually dimorphic in many species of mammals (Tease and Hulten, 2004; Lenormand and Dutheil, 2005). Females could show evolutionary patterns distinct from those we report for males. Moreover, our characterization of crossover patterning is restricted to chromosome 1, despite the potential for recombination differences among chromosomes. Finally, this study features a modest sample size and may not have sufficient power to detect subtle differences within and between mouse populations and species.

### Conclusions and future research

Even with these caveats, our main conclusion is clear. *P. leucopus* males from the same population recombine at very similar rates (on the genomic scale), position crossovers in similar ways, and show limited divergence in recombination rate between populations and species. Evolutionary modeling—as well as measurements of fitness—will be required to determine whether these patterns of constraint are explained by stabilizing selection. Surveys of recombination rate variation in additional wild populations will be needed to evaluate the generality of our findings.

### Data archiving

Data available from the Dryad Digital Repository: 10.5061/dryad.f2kh311

## Supplementary information

Supplemental Data File 1

Supplementary material legends

Supplementary figure 1

Supplementary figure 2

Supplementary figure 3

Supplementary table 1

Supplementary table 2
