# Supplementary material for: Conservation of the genome-wide recombination rate in white-footed mice
Source: Heredity (Edinb). 2019 Jul 31;123(4):442–57. doi: 10.1038/s41437-019-0252-9 (PMC6781155; doi:10.1038/s41437-019-0252-9)

example_output_whole.tif

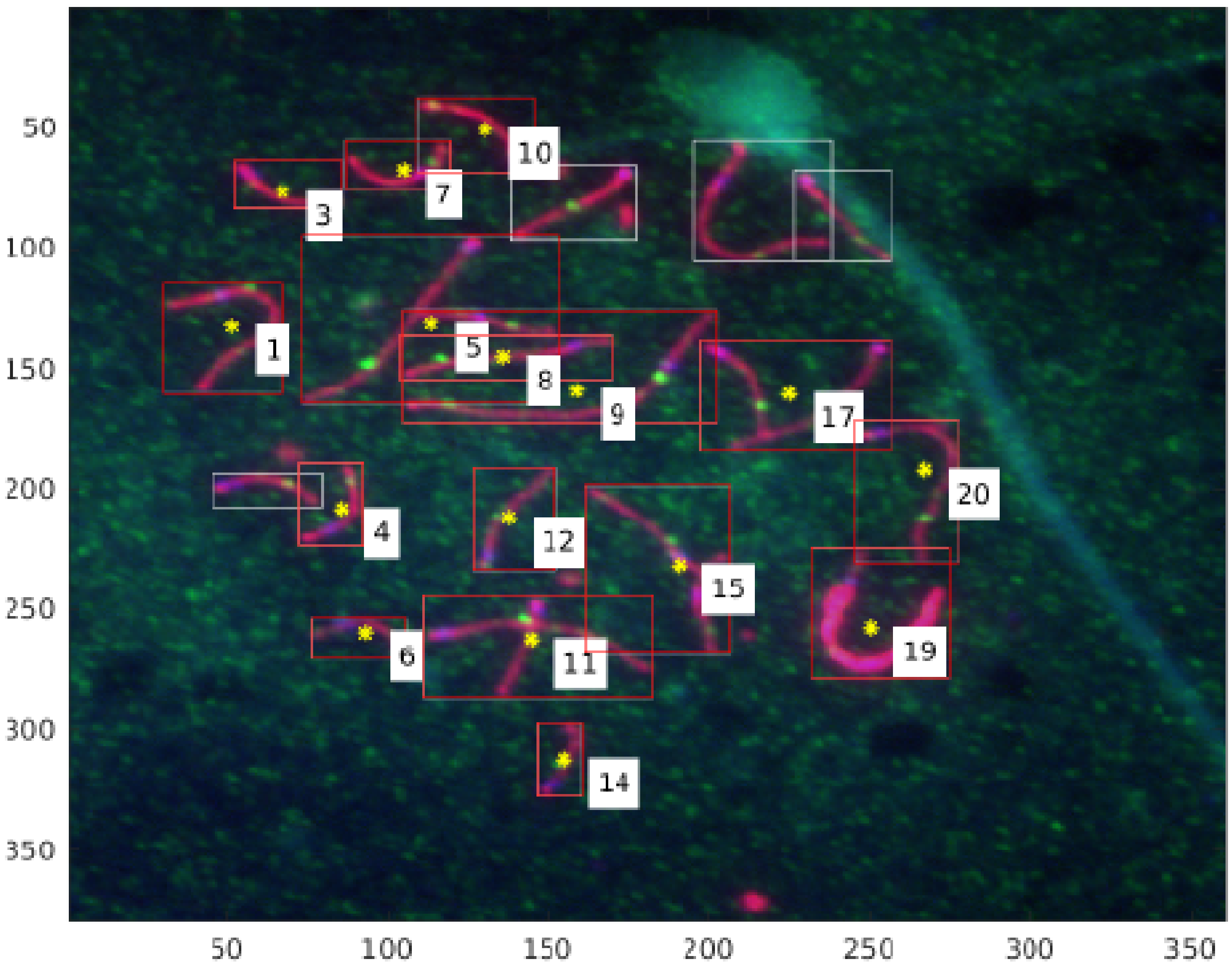

example_output_1_straight2.tif

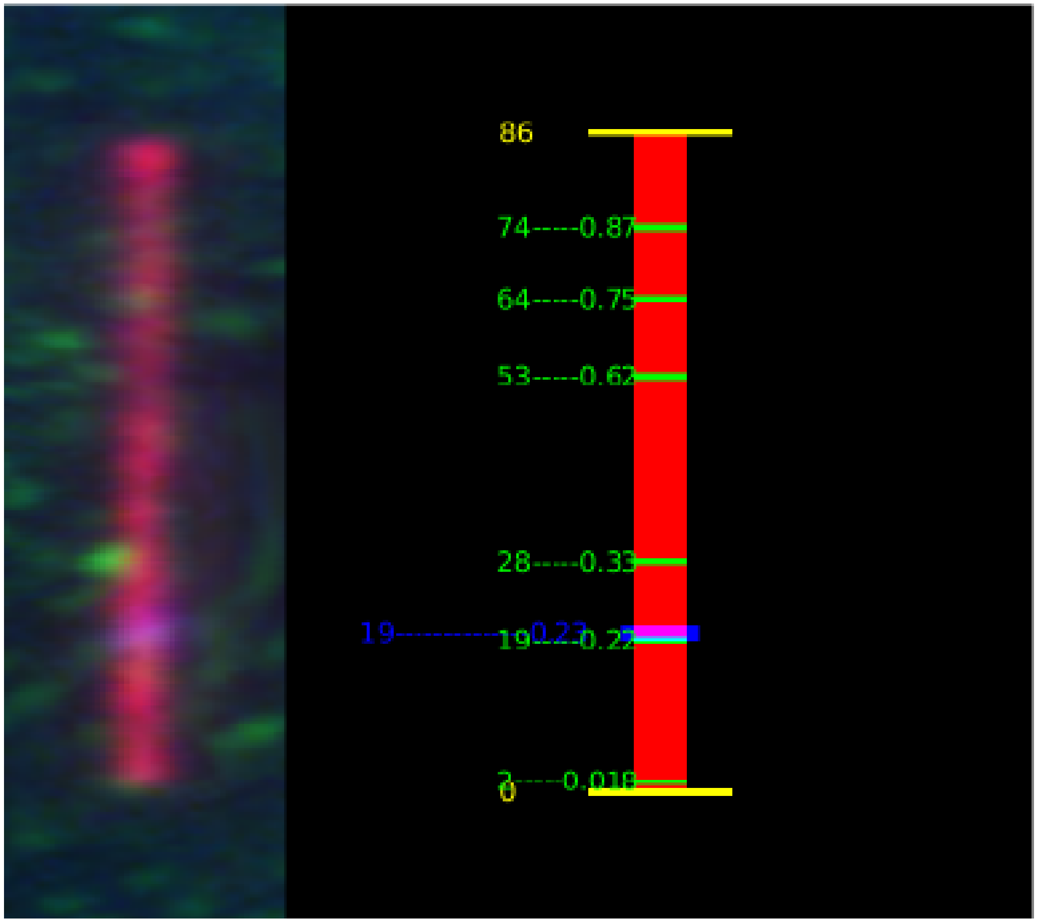

example_output_3_straight2.tif

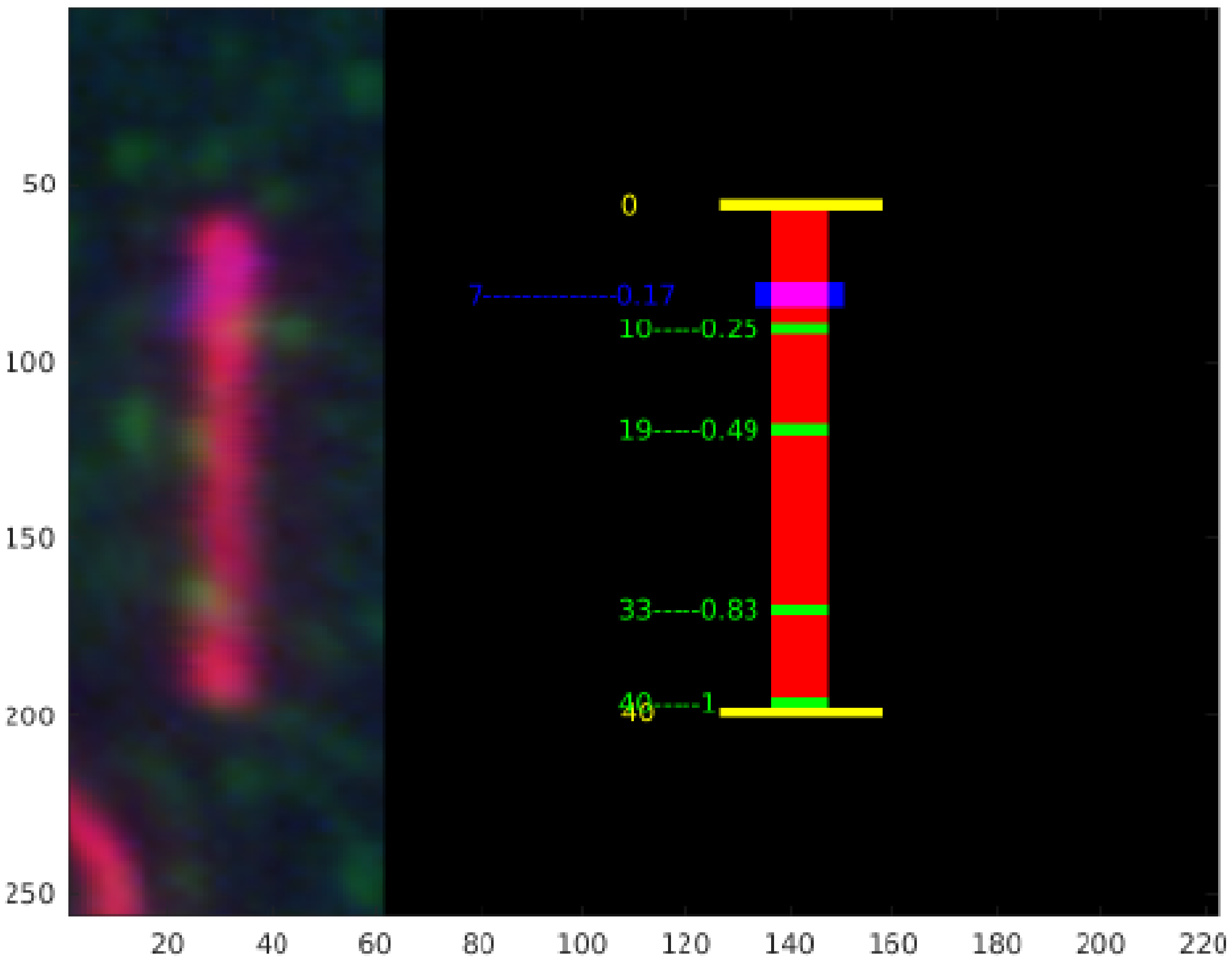

example_output_4_straight2.tif

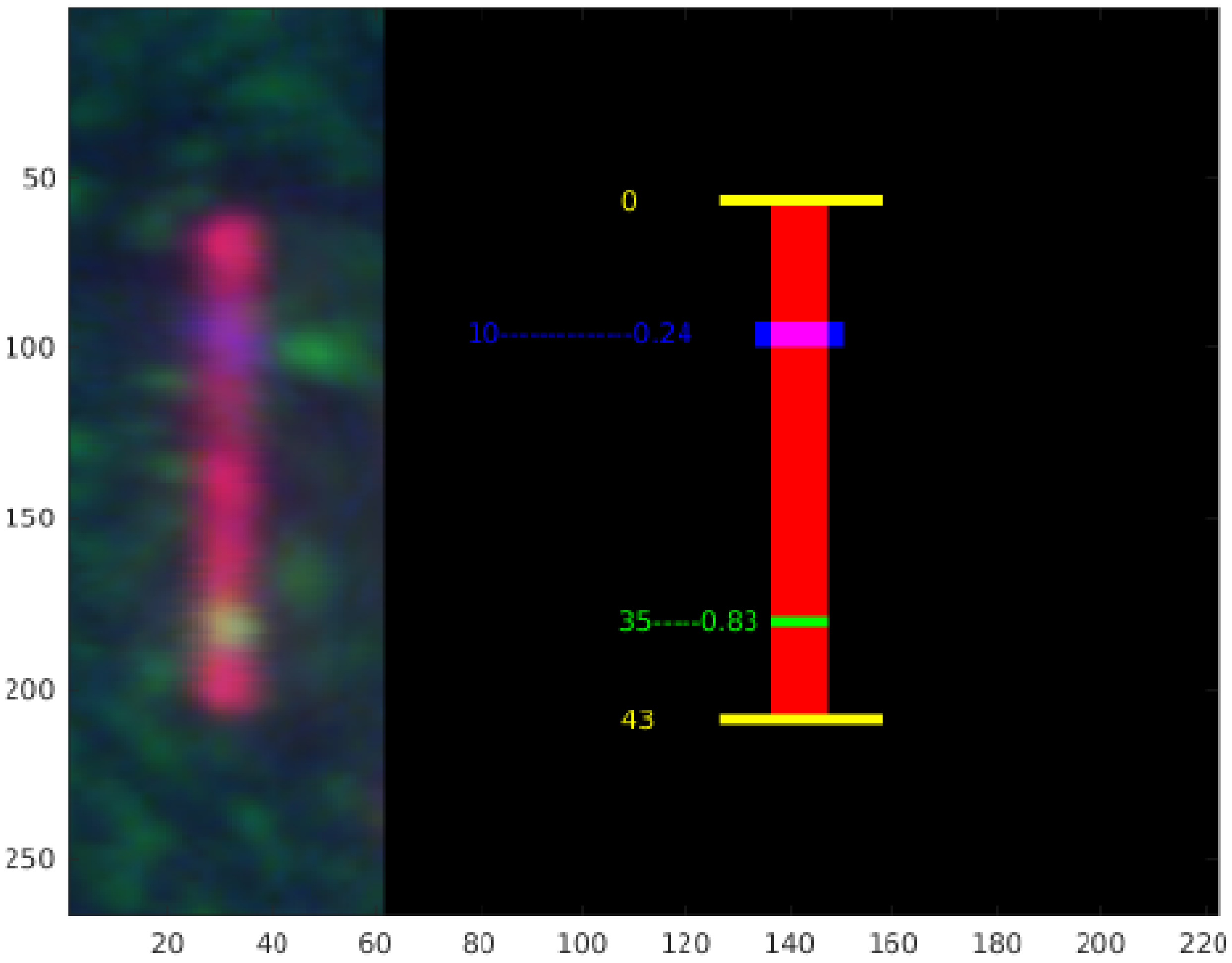

example_output_5_straight2.tif

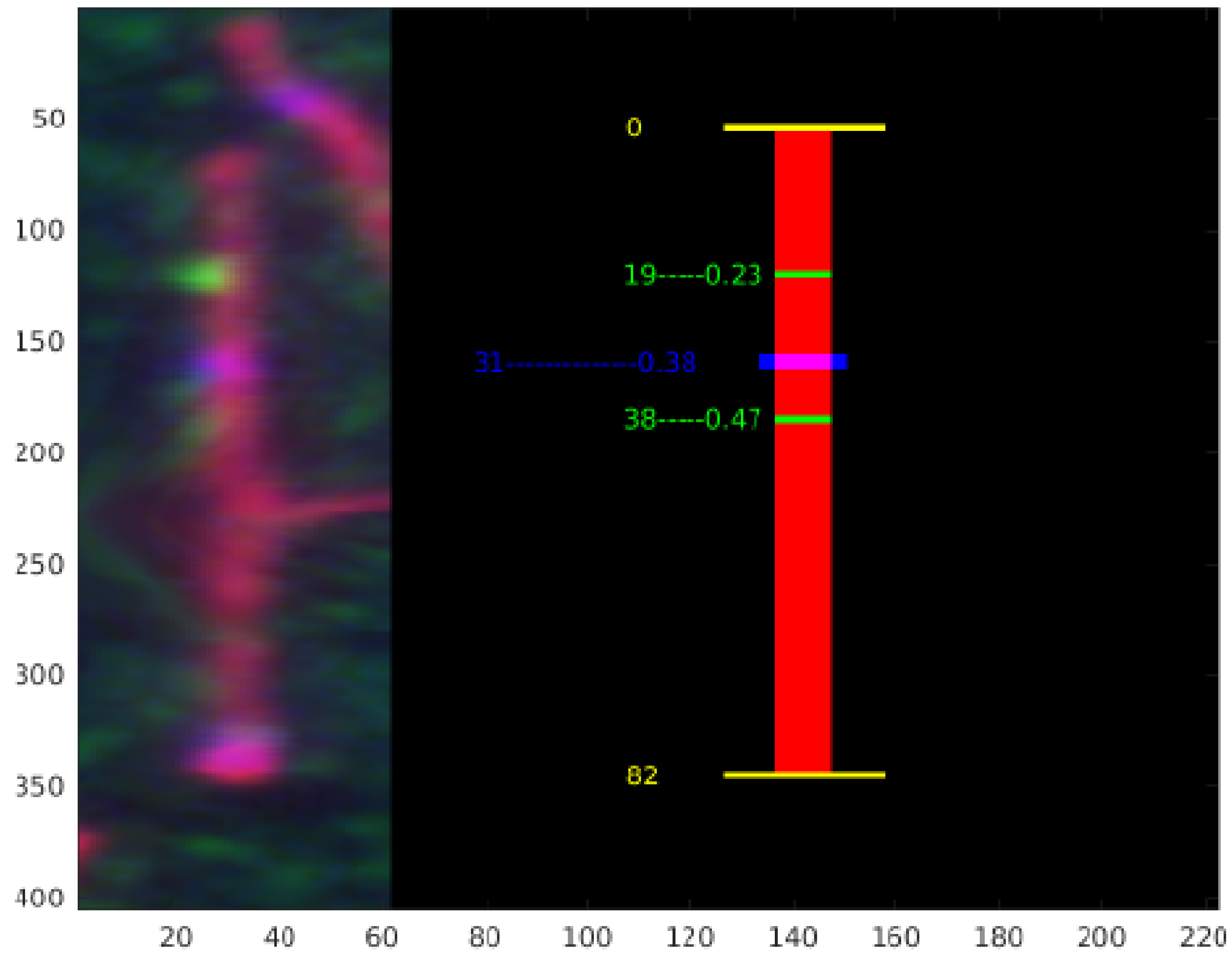

example_output_6_straight2.tif

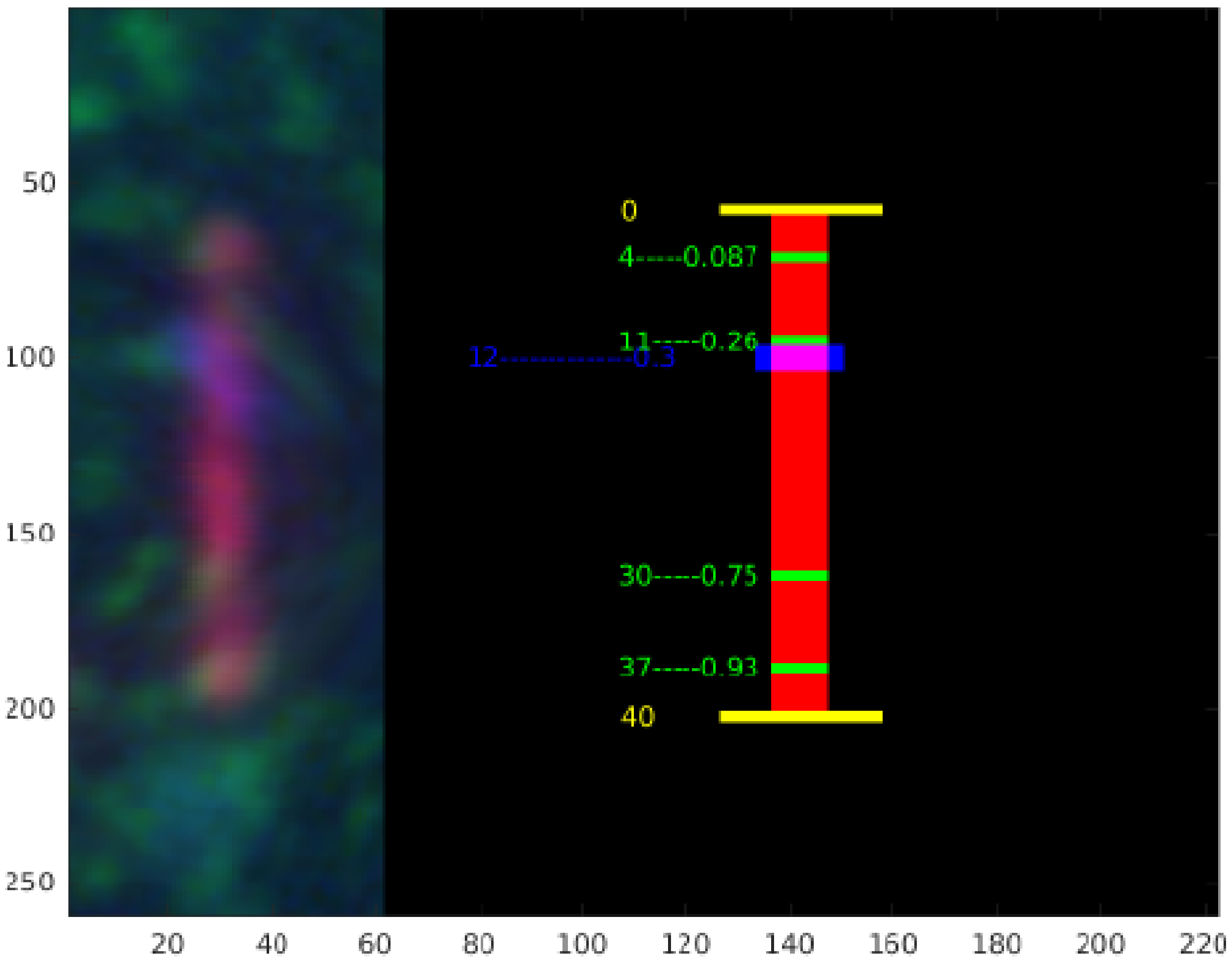

example_output_7_straight2.tif

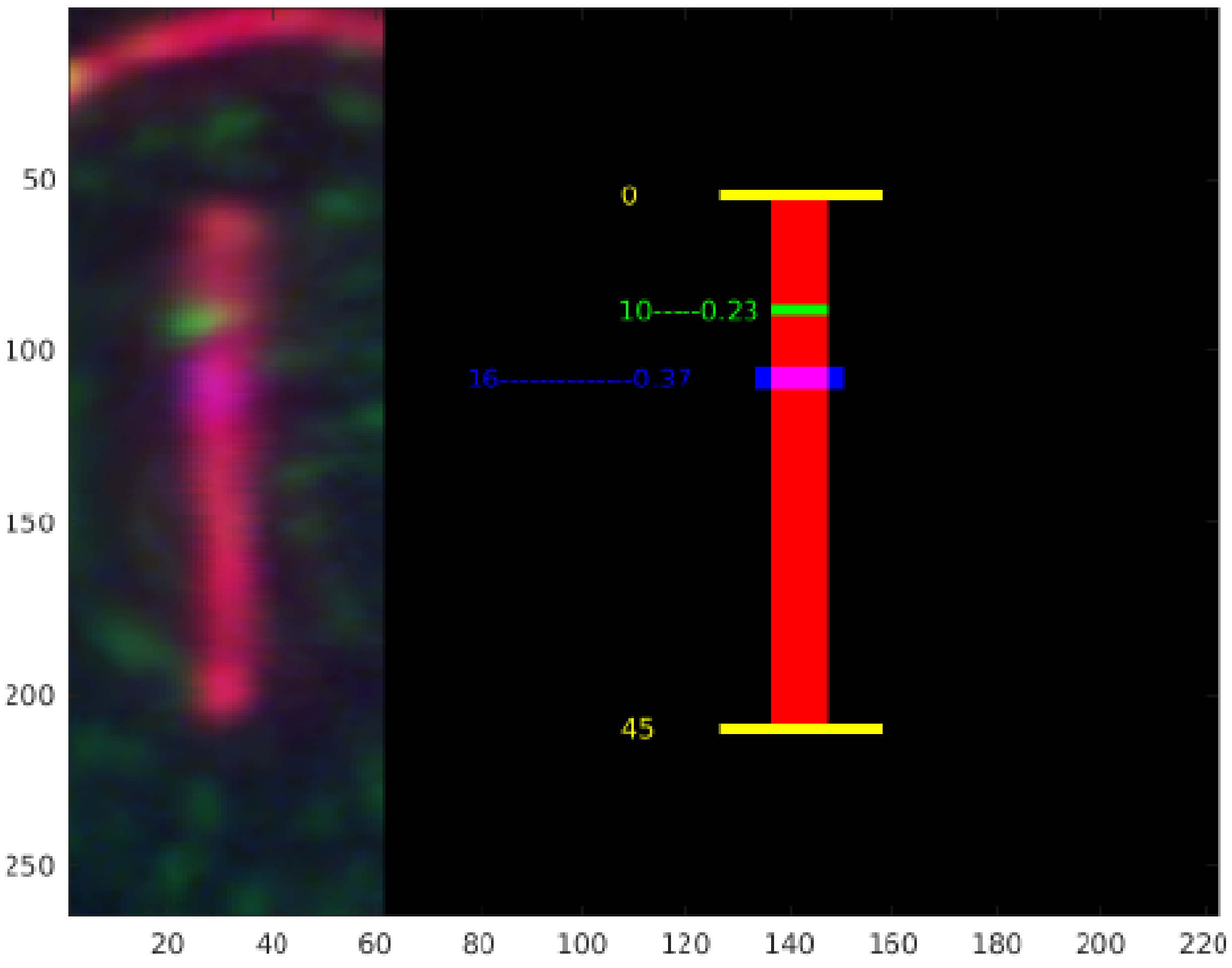

example_output_8_straight2.tif

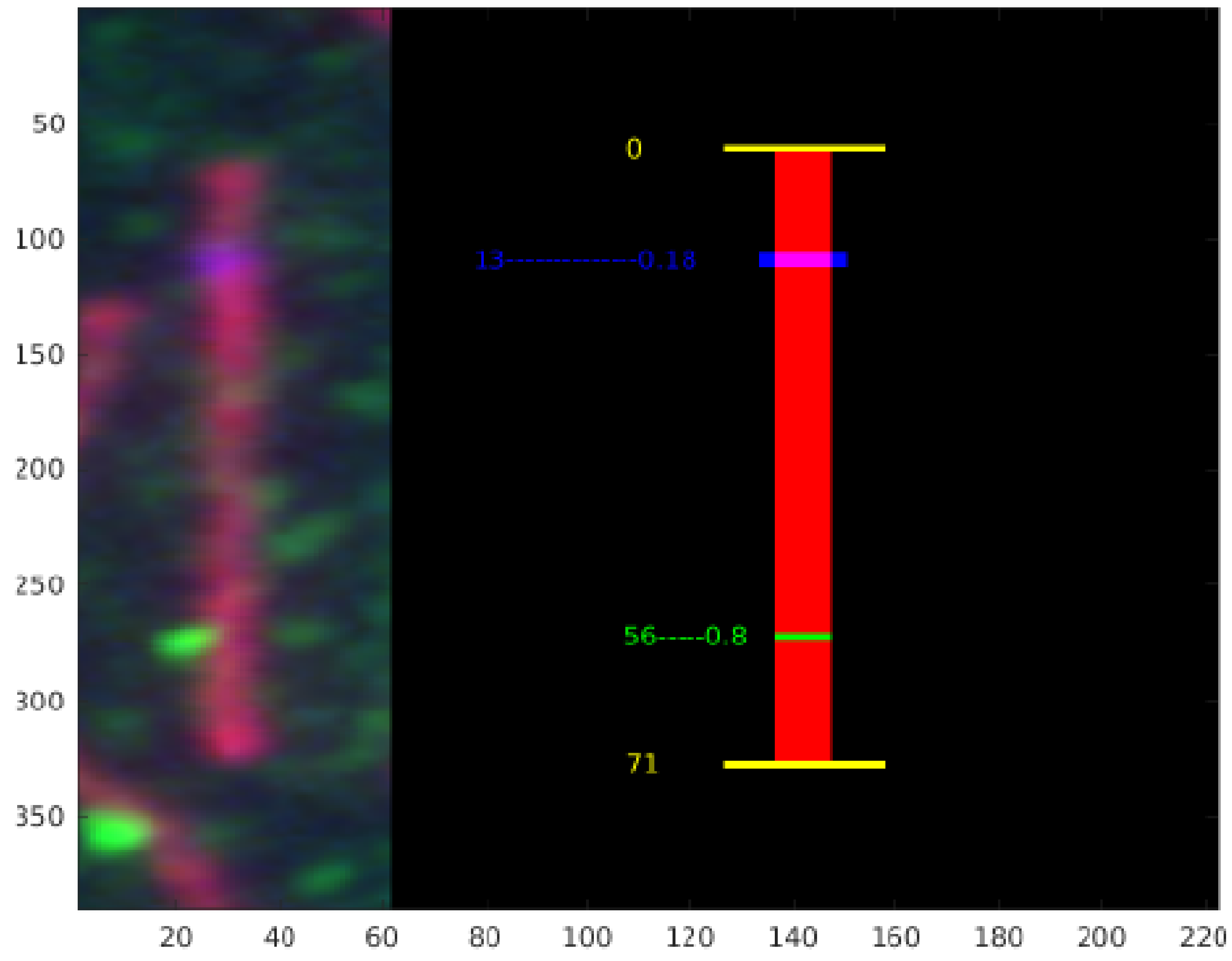

example_output_9_straight2.tif

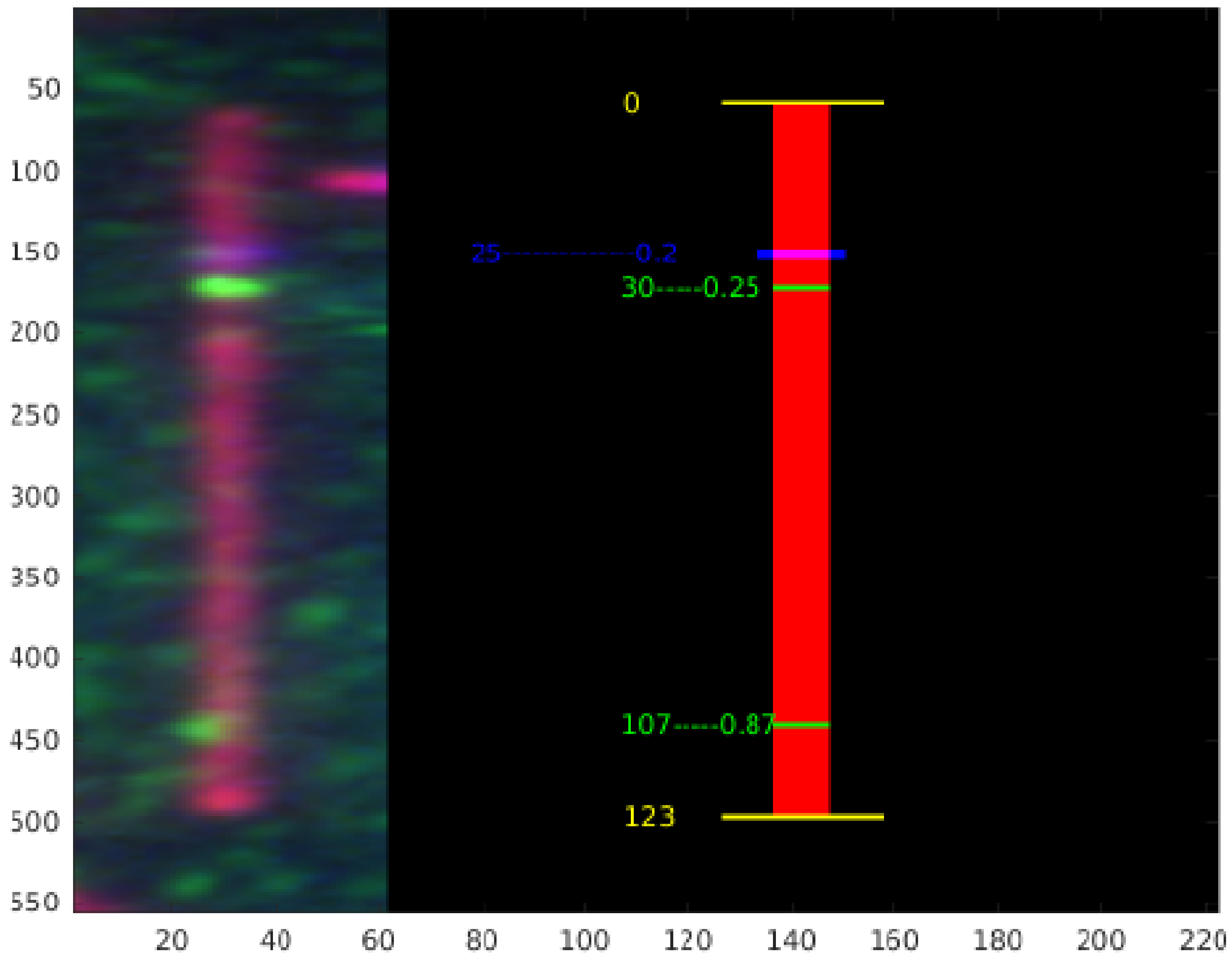

example_output_10_straight2.tif

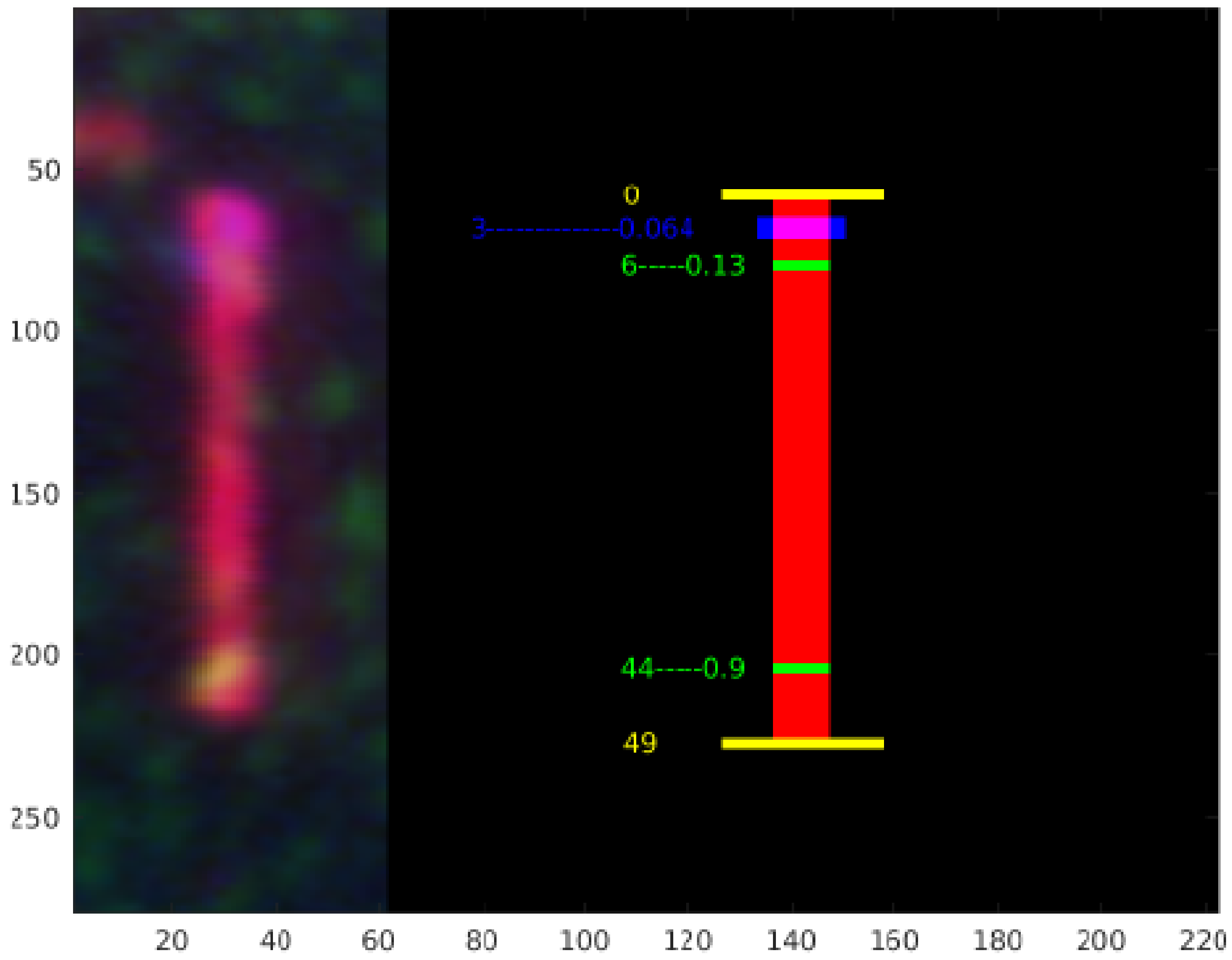

example_output_11_straight2.tif

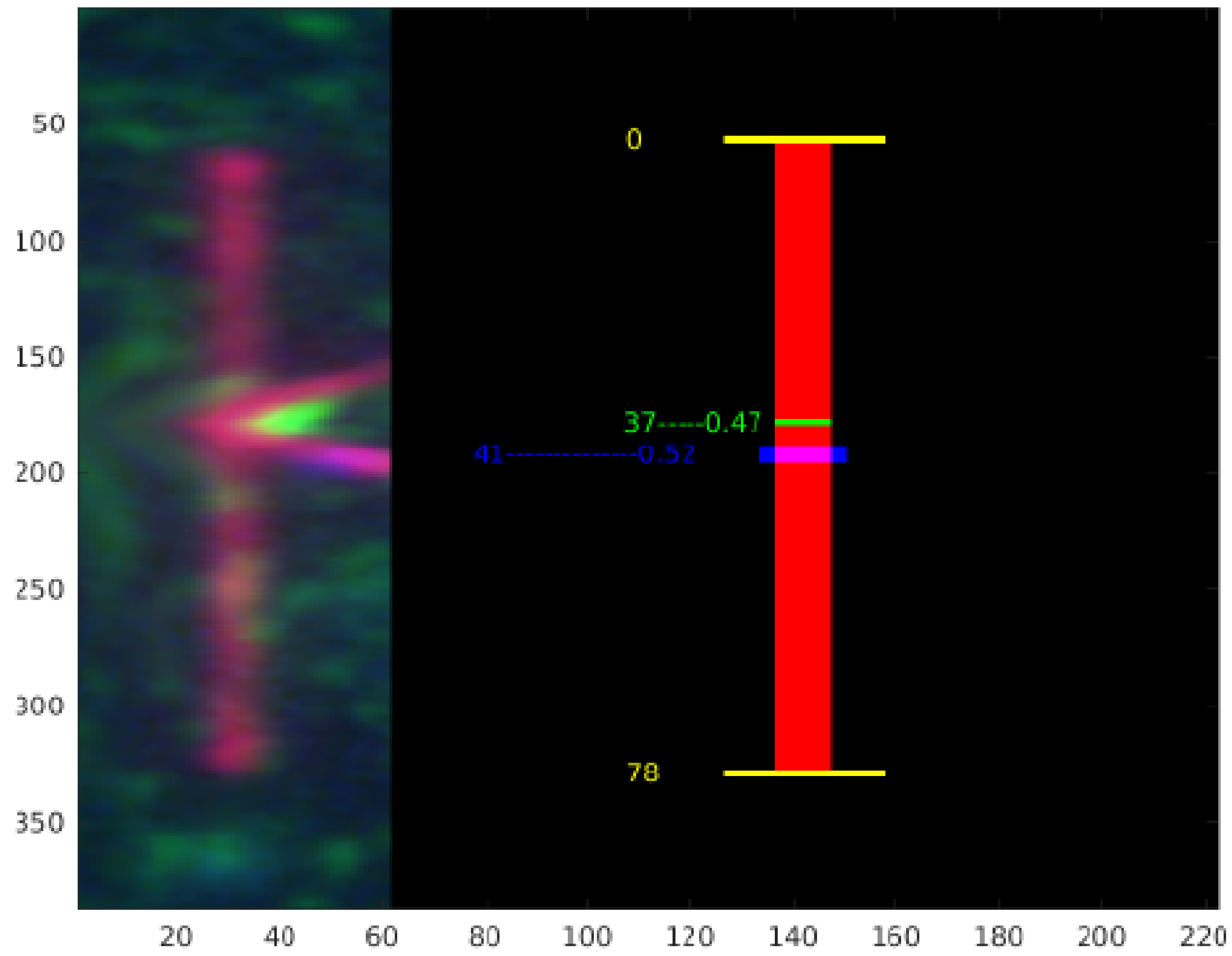

example_output_12_straight2.tif

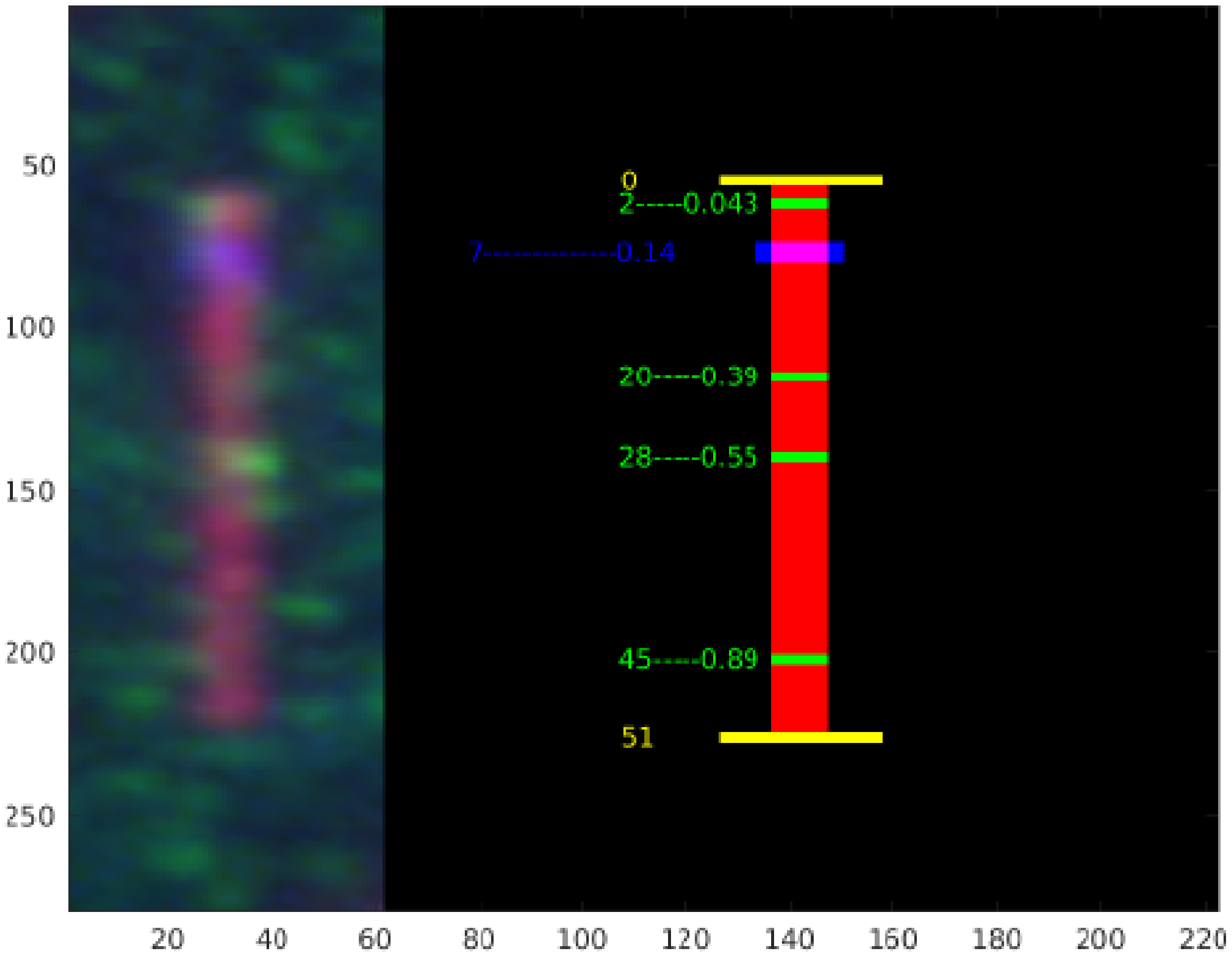

example_output_14_straight2.tif

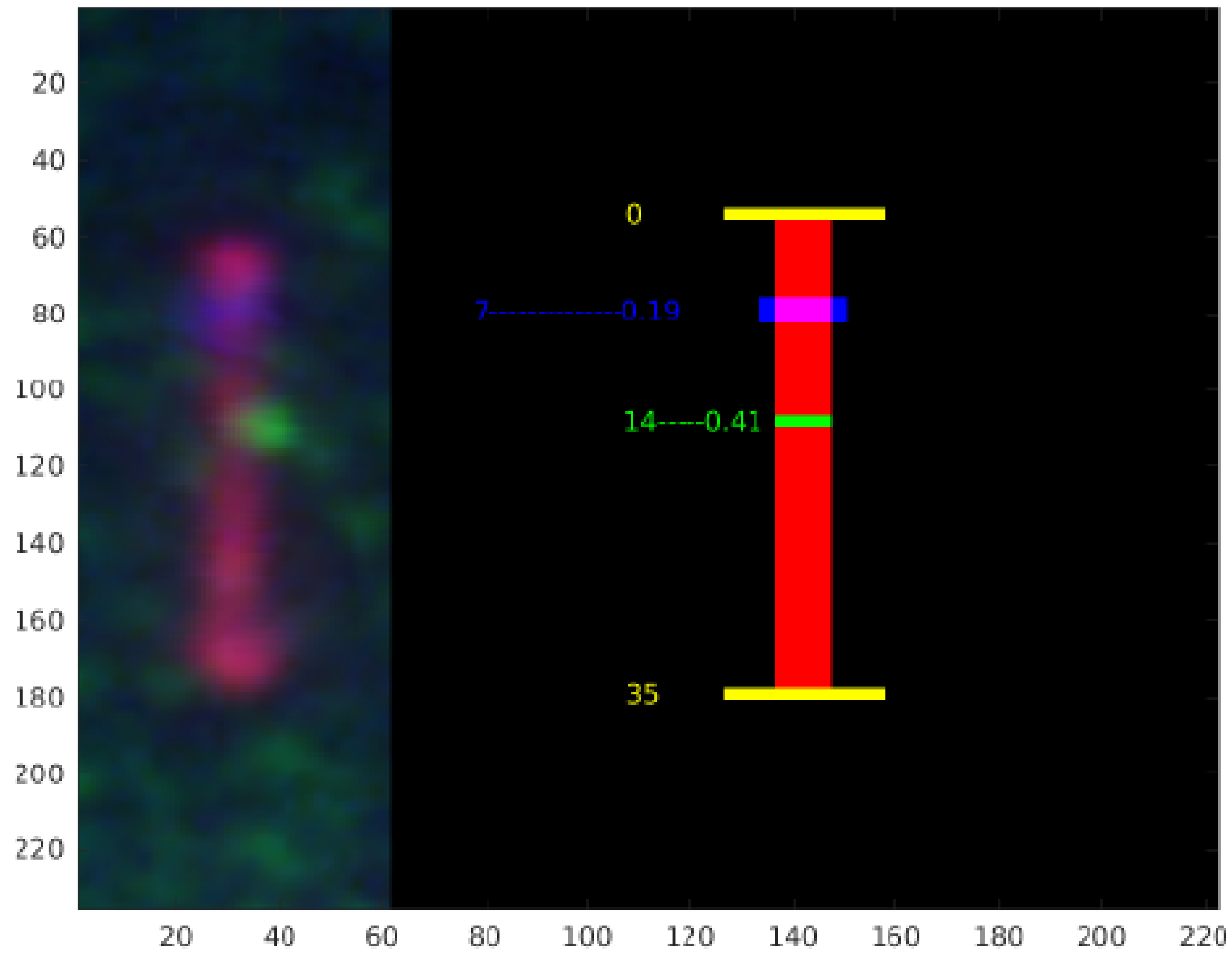

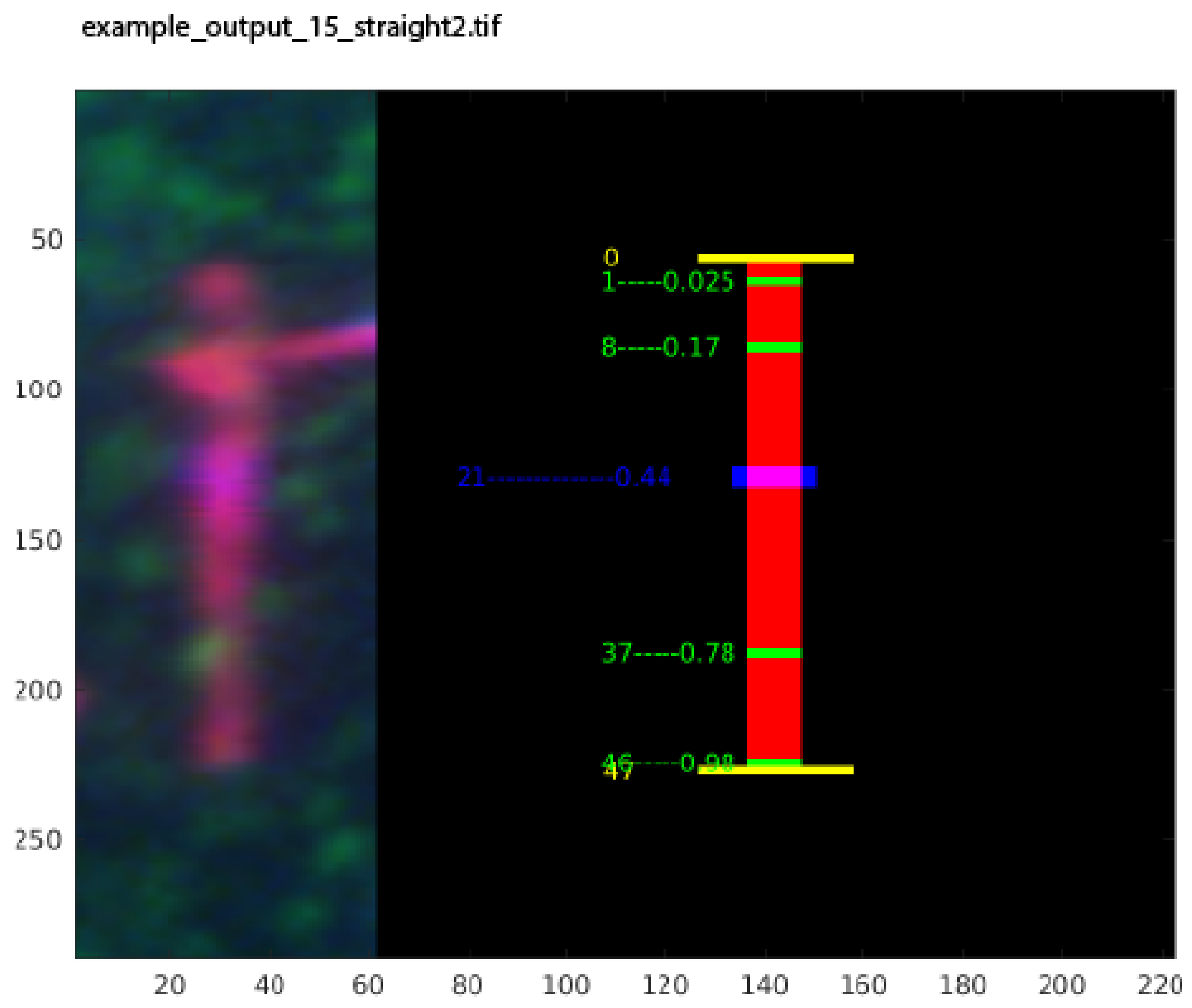
example_output_15_straight2.tif
0
1-----0.025
8-----0.17
21--------------0.44
37-----0.78
47
50
100
150
200
250
20
40
60
80
100
120
140
160
180
200
220

example_output_17_straight2.tif

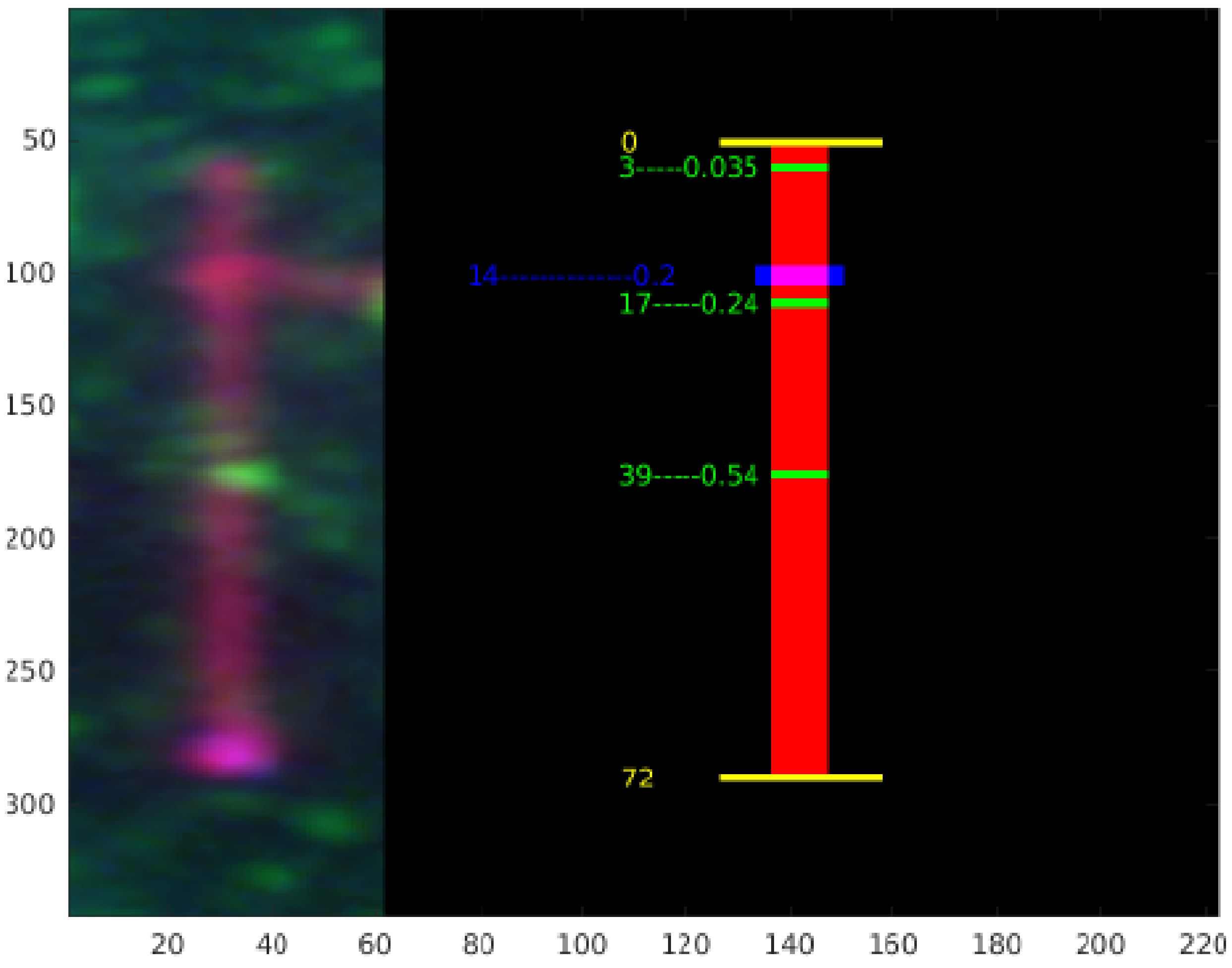

example_output_19_straight2.tif

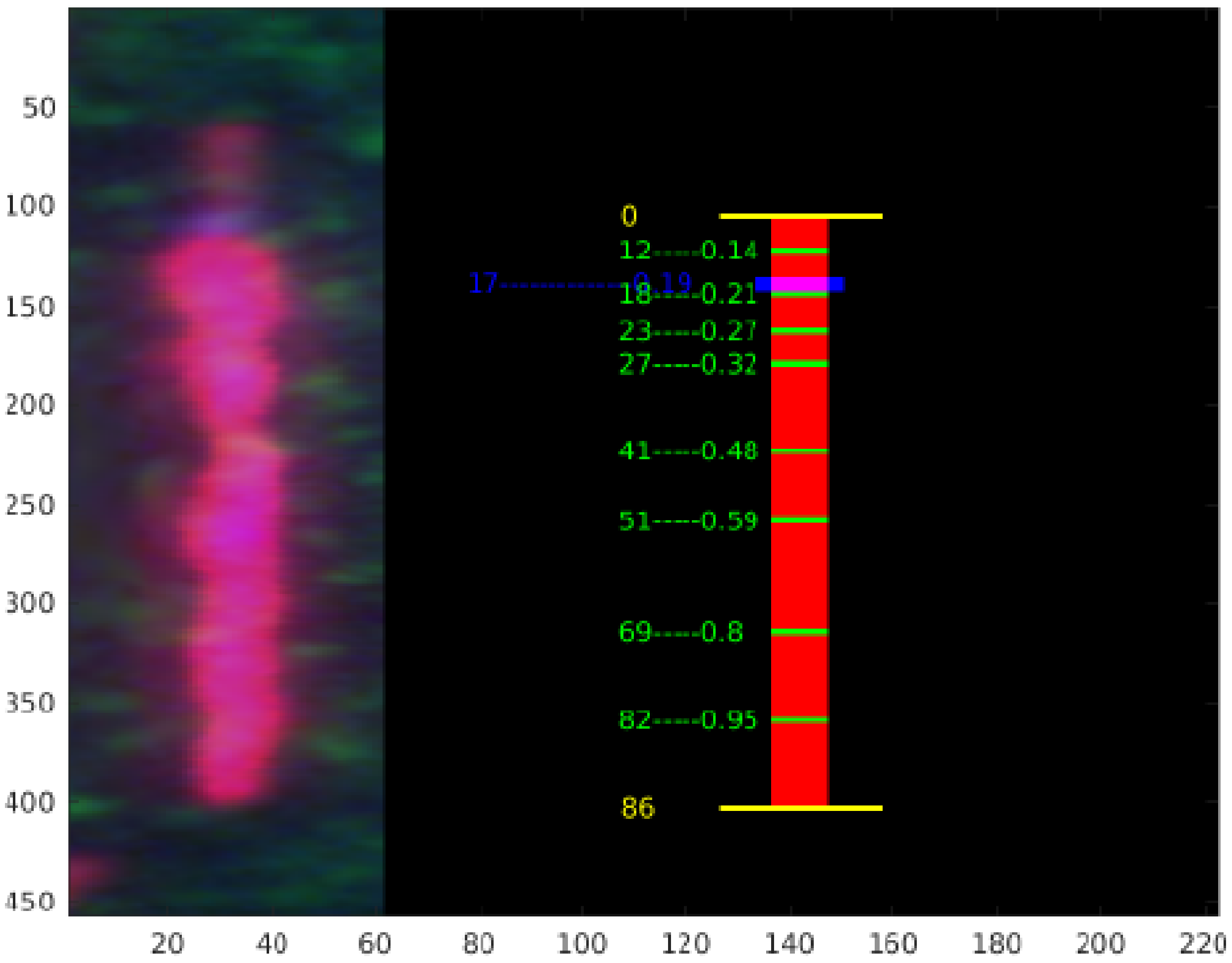

example_output_20_straight2.tif

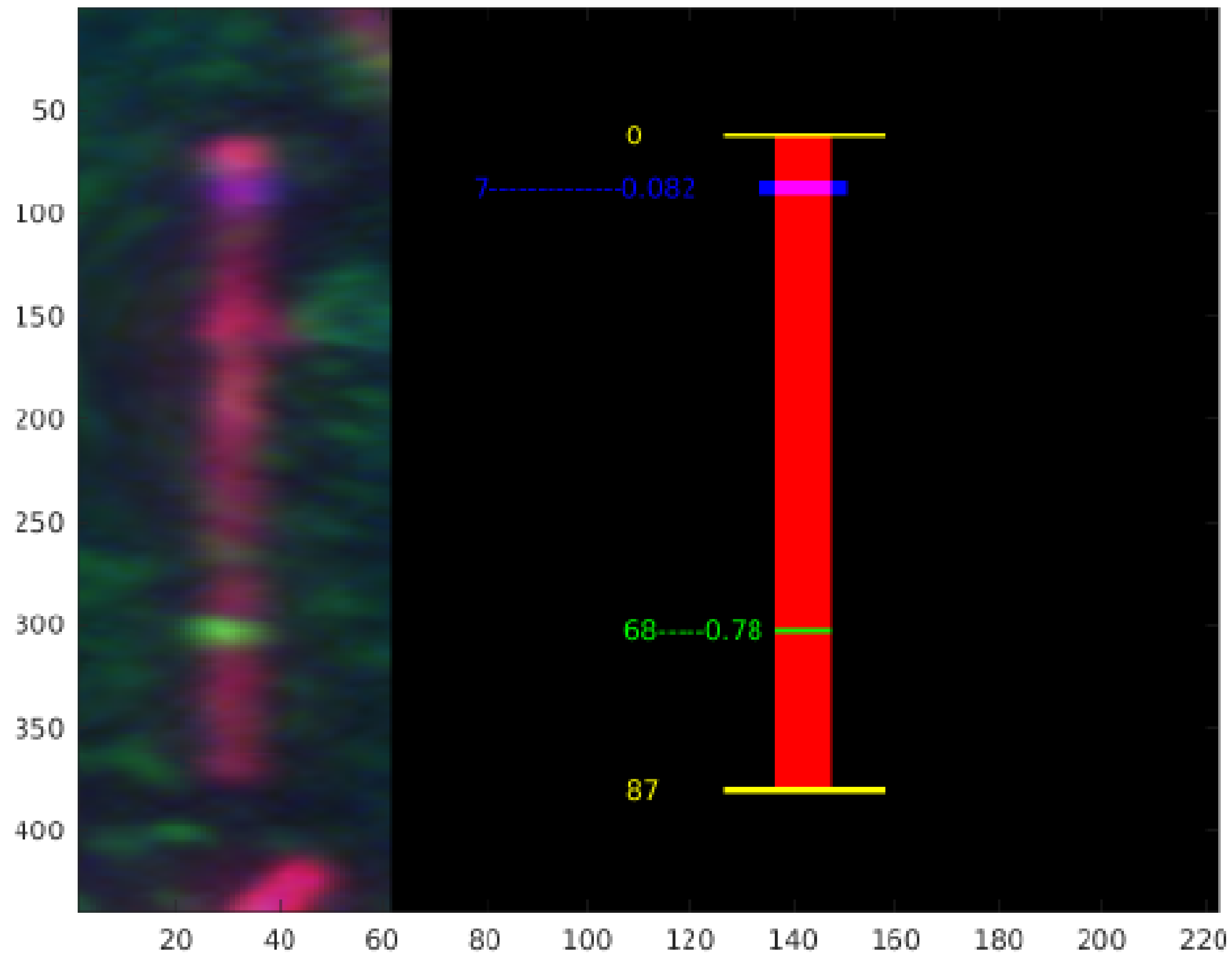

Supplement: Supplementary file 1 — Supplemental Data File 1 [file 41437_2019_252_MOESM1_ESM.pdf]
