# Supplementary material for: Conservation of the genome-wide recombination rate in white-footed mice
Source: Heredity (Edinb). 2019 Jul 31;123(4):442–57. doi: 10.1038/s41437-019-0252-9 (PMC6781155; doi:10.1038/s41437-019-0252-9)
Supplement: Supplementary file 2 — Supplementary material legends [file 41437_2019_252_MOESM2_ESM.docx]

**Supplement Figure 1**

Histograms of MLH1 focus counts across groups of mice.

**Supplement Figure 2**

Boxplots of synaptonemal complex length for wild (A) and laboratory (B) *P. leucopus*. Boxplots are overlaid to illustrate distributions across bivalent classes.

**Supplement Figure 3**

SC lengths by month for wild mice. Each point is a chromosome 1 observation and colored by mouse ID.

**Supplemental Table 1**

Wild mouse information. Species, Mouse ID, number of cells used for MLH1 count and chromosome 1 observations listed. Trap date, location and notable features are listed for wild mice listed.

**Supplemental Table 2**

Laboratory-raised mouse information. Species, Mouse ID, number of cells used for MLH1 count and chromosome 1 observations listed. Date of birth and age in week are listed.
