# Supplementary figures and images for: Conservation of the genome-wide recombination rate in white-footed mice
Source: Heredity (Edinb). 2019 Jul 31;123(4):442–57. doi: 10.1038/s41437-019-0252-9 (PMC6781155; doi:10.1038/s41437-019-0252-9)

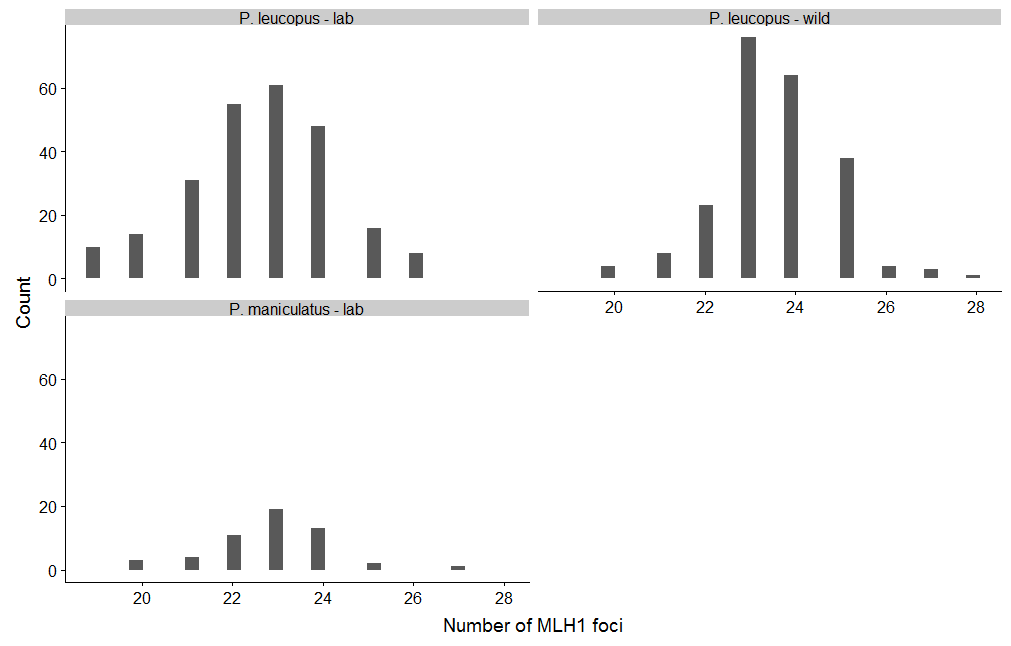

Supplement: Supplementary file 3 — Supplementary figure 1 [file 41437_2019_252_MOESM3_ESM.tif]

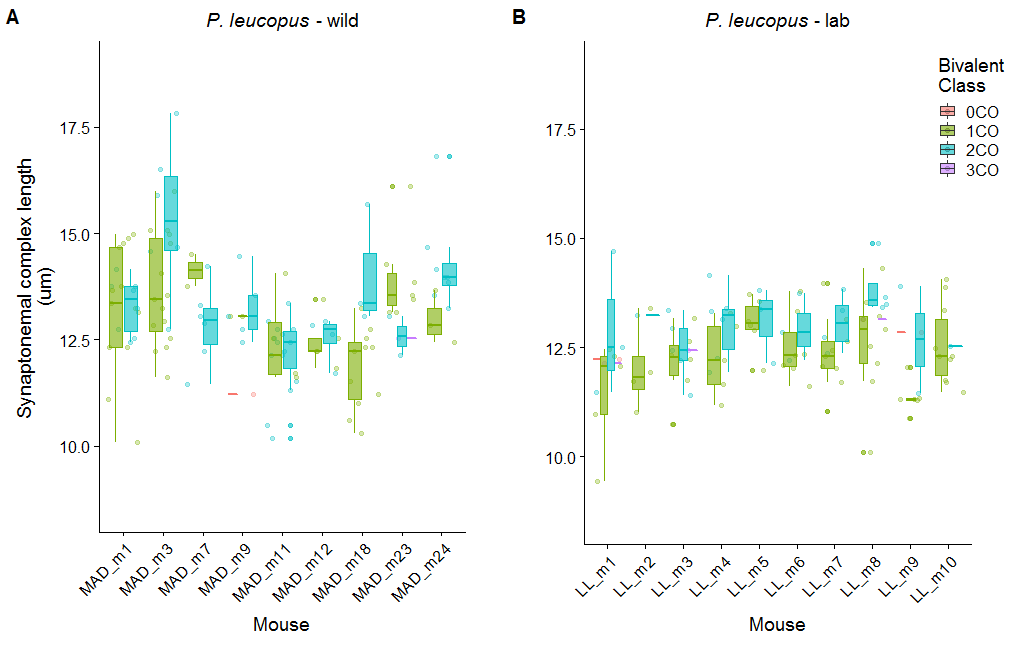

Supplement: Supplementary file 4 — Supplementary figure 2 [file 41437_2019_252_MOESM4_ESM.tif]
